# TSPO is a potential independent prognostic factor associated with cellular respiration and p16 in head and neck squamous cell carcinoma

**DOI:** 10.3389/fonc.2023.1298333

**Published:** 2023-12-14

**Authors:** Sanni Tuominen, Linda Nissi, Antti Kukkula, Johannes Routila, Teemu Huusko, Ilmo Leivo, Heikki Minn, Heikki Irjala, Eliisa Löyttyniemi, Sami Ventelä, Maria Sundvall, Tove J. Grönroos

**Affiliations:** ^1^Preclinical Imaging Laboratory, Turku PET Centre, University of Turku, Turku, Finland; ^2^Cancer Research Unit, Institute of Biomedicine, University of Turku, Turku, Finland; ^3^FICAN West Cancer Research Laboratory, Turku University Hospital and University of Turku, Turku, Finland; ^4^Medicity Research Laboratory, Faculty of Medicine, University of Turku, Turku, Finland; ^5^Department of Clinical Oncology, Turku University Hospital and University of Turku, Turku, Finland; ^6^Department of Otorhinolaryngology – Head and Neck Surgery, Turku University Hospital and University of Turku, Turku, Finland; ^7^Department of Pathology, Turku University Hospital and University of Turku, Turku, Finland; ^8^Department of Biostatistics, Turku University Hospital and University of Turku, Turku, Finland

**Keywords:** head and neck cancer, biomarker, p16, translocator protein, oxidative phosphorylation, immune landscape

## Abstract

**Background:**

Treatment resistance and relapse are common problems in head and neck squamous cell carcinoma (HNSCC). Except for p16, no clinically accepted prognostic biomarkers are available for HNSCC. New biomarkers predictive of recurrence and survival are crucial for optimal treatment planning and patient outcome. High translocator protein (TSPO) levels have been associated with poor survival in cancer, but the role of TSPO has not been extensively evaluated in HNSCC.

**Materials and methods:**

TSPO expression was determined in a large population-based tissue microarray cohort including 611 patients with HNSCC and evaluated for survival in several clinicopathological subgroups. A TCGA HNSCC cohort was used to further analyze the role of *TSPO* in HNSCC.

**Results:**

TSPO expression was downregulated in more aggressive tumors. Low TSPO expression associated with worse 5-year survival and was an independent prognostic factor for disease-specific survival. Subgroup analyses showed that low TSPO expression associated with worse survival particularly in p16-positive oropharyngeal cancer. *In silico* analyses supported the prognostic role of *TSPO.* Cellular respiration had the highest significance in pathway analyses for genes expressed positively with *TSPO.*

**Conclusion:**

Decreased TSPO expression associates with poor prognosis in HNSCC. TSPO is a prognostic biomarker in HNSCC to potentially guide treatment stratification especially in p16-positive oropharyngeal cancer.

## 1 Introduction

Head and neck squamous cell carcinoma (HNSCC) is a heterogeneous group of tumors originating in the head and neck region (1, 2). HNSCC is the seventh most common cancer worldwide (3) and survival rates vary depending on factors, such as human papillomavirus (HPV) status, anatomical tumor site, and disease stage (4). Patients with an early stage I/II disease have 5-year survival rates of 70–90%, whereas less than 50% of patients with an advanced stage III/IV disease will survive (1, 2, 5).

Local HNSCC is treated with either surgery or radiotherapy with or without chemotherapy or with a combination of surgery and radiotherapy with or without chemotherapy (1, 5). Metastatic disease is mostly treated with platinum-based chemotherapy, EGFR-targeting antibody cetuximab, or PD-1–targeting immune checkpoint inhibitors (4, 6). Although advancements in treatment modalities have been made, the disease outcome has not improved significantly mainly due to treatment resistance and cancer recurrence (5, 7). In addition, standard treatment strategies are toxic and significantly decrease patient quality of life (7).

Several studies have shown a favorable prognosis of HPV-associated HNSCC (8, 9) and hence lower TNM staging for HPV-positive tumors was adopted in the 8^th^ edition of the TNM classification of HNSCC (10). p16 expression is widely used as a surrogate marker for HPV in oropharyngeal cancer. Even though p16-positive tumors respond, for a yet unknown reason, better to chemotherapy and radiotherapy (9, 11, 12), and p16 is an independent prognostic factor, its role in treatment stratification remains unclear (13). In addition, PDL-1 is currently being evaluated as a predictive marker for immune checkpoint therapy for recurrent or metastatic disease (14, 15). Biomarkers to guide clinical decision making and new therapeutic targets in HNSCC are urgently needed.

TSPO (translocator protein, also known as PBR), is an 18-kDa protein located mainly on the mitochondrial outer membrane (16, 17). TSPO interacts with VDAC (voltage-dependent anion channel) and ANC (adenine nucleotide carrier), but it also functions alone as a monomer, dimer, or oligomer (18–21). TSPO participates in a wide range of cellular functions, such as cholesterol transport and steroidogenesis, MPTP (mitochondrial permeability transition pore) regulation, reactive oxygen species homeostasis, apoptosis, autophagy, inflammation, and porphyrin transport (17, 21–23). However, studies with TSPO knockout mice showing no changes in cholesterol transport, steroidogenesis (24–28), and mitochondrial permeability transition (29) have questioned the importance of TSPO in these functions. Altogether, the functions and physiological role of TSPO are still not completely understood (17, 23).

Several studies have shown that TSPO expression is elevated in cancer and correlate with poor survival in glioblastoma (30, 31), breast cancer (32, 33), prostate cancer (33, 34), liver cancer (35, 36), colorectal cancer (33, 37, 38), melanoma (39), and esophageal squamous cell carcinoma (40). However, there is relatively little information on the possible prognostic role of TSPO in HNSCC. High TSPO expression has been correlated with poor survival in a small cohort of patients with oral squamous cell carcinomas (41). Due to the heterogeneous nature of HNSCC, further studies with larger cohorts, including tumors originating from different anatomical sites of the head and neck region, are needed.

As there is an urgent need for improved biomarkers to guide treatment decision making, we used a large primary HNSCC population-based cohort, including comprehensive follow-up data of patients, to investigate the association between TSPO expression and survival with clinicopathological parameters. TSPO expression was also analyzed in different primary tumor subsites and according to p16 status. Moreover, *in silico* database analyses were performed to further study the functional role of TSPO in different biological processes and pathways in HNSCC, and as a modulator of immune landscape of tumors.

## 2 Methods

### 2.1 HNSCC patient cohort tissue microarray

The HNSCC cohort has been previously described (42–44). Briefly, the population-based cohort consisted of all patients newly diagnosed with HNSCC and treated at Southwest Finland regional referral center of Turku University Hospital from 2005 to 2015. In total, 1033 patients were included in the cohort, of which 611 samples were available for immunohistochemical (IHC) staining with an antibody against TSPO. The usage of human tissue samples was approved by the institutional Review Board of the Finnish national authority for medicolegal affairs (V/39706/2019), regional ethics committee of University of Turku (51/1803/2017), and Auria Biobank scientific board (AB19-6863). The authors affirm that the study was conducted following the rules of the Declaration of Helsinki of 1975, revised in 2013. Informed consent was waived due to the retrospective design of the study according to the Finnish Act on Secondary Use of Social and Health Data effective from April 2019 (Act 552/2019). All data were collected, stored, and handled in a manner that meets the regulation of GDPR and the Secondary Use Act 552/2019.

Overall survival (OS) was determined as the end-of-treatment to end-of-follow-up or death. Disease-specific survival (DSS) was determined as the end-of-treatment to end-of-follow-up or death from HNSCC. Disease-free survival (DFS) was determined as the end-of-treatment to disease progression. Patients with 0 survival days were excluded from the DFS analysis.

### 2.2 IHC

TMA blocks with duplicate 0.6 mm core biopsies were made from formalin-fixed, paraffin-embedded tissue samples using a TMA Grand Master (3DHISTECH, Budapest, Hungary) at Auria Biobank (Turku, Finland). After optimization (**Supplementary Figure S1**), IHC staining was performed with a Labvision autostainer (Thermo Fisher Scientific, Waltham, MA, USA). Briefly, after deparaffination and rehydration, endogenous peroxidase activity was blocked with hydrogen peroxide at room temperature. Antigen retrieval was carried out with citrate buffer and microwaving. The sections were then incubated with a TSPO antibody (rabbit monoclonal, 1:50 000, ab109497, Abcam, Cambridge, UK) for 60 min at room temperature. We have previously validated this antibody with *TSPO*-targeting siRNAs by Western blotting (45). Primary antibody visualization was done with a secondary goat anti-rabbit HRP antibody (BrightVision 2 steps detection system, DPVB110HRP, WellMed, Duiven, the Netherlands) for 30 min. After 3,3-diaminobenzidine reaction (BrightDAB, BS04-110, WellMed), the sections were counterstained with Mayer’s hematoxylin.

IHC staining was independently analyzed by two authors (ST, LN). Any inconsistent findings were discussed with a third author (SV) to reach a consensus. Cytoplasmic TSPO expression was scored semi-quantitatively based on staining intensity on a scale of 0–3. For statistical analysis, staining intensities were grouped as either low (scores 0 and 1) or high (scores 2 and 3). The p16 staining was performed and analyzed previously (44).

### 2.3 *In silico* analysis

For *in silico* analyses, *TSPO* expression from the Cancer Genome Atlas Program (TCGA) Head and Neck Cancer (HNSCC) patient dataset (TCGA Data Coordinating Center in Jan 2016 and Broad Firehose analyses 2016-01-28) was acquired from the UCSC Xena database (46). *TSPO* expression (IlluminaHiSeq_RNASeqV2, version 2017-10-13) was available from 566 patients with a median expression value of 11.965 [log2(norm_count+1)] in primary tumor samples (n = 520). OS, DSS, and progression-free interval (version 2018-09-13) was available from 520 patients, and disease-free interval (version 2018-09-13) was available from 130 patients. The number of patients included in subgroup analyses and the data version used for the analyses are shown in **Supplementary Table S1**.

Additional *in silico* analysis of TSPO expression on the protein level was performed utilizing the Clinical Proteomics Tumor Analysis Consortium (CPTAC) pan-cancer HNSCC cohort (47). TSPO proteomics (Log2 MS1 intensity) data was acquired from LinkedOmics (48).

To study the functional role of TSPO in HNSCC, co-expression analyses between *TSPO* and other genes were performed with cBioPortal (49, 50) using the TCGA HNSCC PanCancer Atlas (51) patient cohort. A list of co-expressing genes with *TSPO* [RSEM (batch normalized from Illumina HiSeq_RNASeqV2)] was available from 488 patients. The total number of genes in the list was 20 058. The top 100 genes (FDR < 0.05) with the highest positive (**Supplementary Table S2**) or negative (**Supplementary Table S3**) expression with *TSPO* were selected for gene overlap analyses with the Molecular Signatures Database (MSigDB, v7.0) (52–54). The Hallmark (53), Kyoto Encyclopedia of Genes and Genomes Canonical Pathways (CP : KEGG) (https://www.kegg.jp/kegg/), and Gene Ontology Biological Process (GO : BP) (http://geneontology.org/) gene sets were selected.

The correlation between *TSPO* expression and the abundance of immune cells in different cancer types was analyzed using TISIDB, which is an integrated repository portal for tumor–immune system interactions (55). TISIDB uses the data from TCGA datasets and infers the relative abundance of immune cell types (56) by using gene set variation analysis based on the gene expression profiles.

### 2.4 Statistical analysis

Statistical analyses were carried out with IBM SPSS Statistics for Windows version 28 (IBM Corp., Armonk, NY, USA). The chi-square test of independence was used to determine the difference in the frequency of low and high TSPO expression in groups. The Cox’s proportional hazard model was used for Kaplan–Meier survival and hazard ratio (HR) with 95% confidence interval (CI) analyses. For *in silico* analyses, primary tumor sites were grouped into oral cavity, larynx, tonsil, hypopharynx/oropharynx, tongue, and floor of the mouth. Because of a low number of samples in the rest of the primary tumor sites, these were combined into a single subgroup named ‘Other’. For all analyses, tumor size (T) classification was divided into low (T1–T2) or high (T3–T4 + TX), and nodal status (N) classification was divided into no lymph node metastasis (N0) or lymph node metastasis (N+). The independent t-test was used to compare *TSPO* mRNA and TSPO protein expression between two groups and one-way ANOVA followed by Bonferroni *post hoc* tests was used when comparing multiple groups. p-values from the hypergeometric distribution and false discovery rate q-values for gene overlap analyses were acquired from MSigDB. TISIDB was used to analyze the correlation (Spearman’s rank correlation coefficient) between *TSPO* expression and immune cell abundance in different cancers. p-values < 0.05 (two-tailed) were considered statistically significant.

## 3 Results

### 3.1 Patient characteristics

Patient characteristics such as gender, age, primary tumor site, T classification, N classification, disease stage, histological grade (G), and p16 status of the TMA cohort are shown in **Table 1**. The most common primary tumor site was the oral cavity (48.4%), followed by oropharynx (23.9%) and larynx (15.7%). Due to the low number of hypopharyngeal (n = 27), nasopharyngeal (n = 24), sinonasal (n = 5), and unknown (n = 17) primary tumor sites, these groups were combined as ‘Other’ (11.9%).

**Table 1:** Patient characteristics and expression frequencies of low vs. high TSPO in different subgroups in the TMA patient cohort.

		Total		Low TSPO^a^		High TSPO^a^		Low vs. high TSPO	
		No. of patients	%	No. of patients	%	No. of patients	%	Chi-square	p-value
**Gender**	Male	391	64%	144	61.3%	247	65.7%	1.2	0.269
	Female	220	36%	91	38.7%	129	34.3%		
	Total	611	100%	235	100%	376	100%		
**Age (y)**	<65	288	47.1%	121	51.5%	167	44.4%	2.9	0.088
	≥65	323	52.9%	114	48.5%	209	55.6%		
	Total	611	100%	235	100%	376	100%		
**Site**	Oral cavity	296	48.4%	107	45.5%	189	50.3%	6.1	0.109
	Oropharynx	146	23.9%	58	24.7%	88	23.4%		
	Larynx	96	15.7%	33	14%	63	16.8%		
	Other	73	11.9%	37	15.7%	36	9.6%		
	Total	611	100%	235	100%	376	100%		
**T**	T1–T2	370	62.3%	128	56.1%	242	66.1%	6.0	**0.015**
	T3–T4	224	37.7%	100	43.9%	124	33.9%		
	Total	594	100%	228	100%	366	100%		
**N**	N0	341	56.3%	115	49.1%	226	60.8%	7.9	**0.005**
	N+	265	43.7%	119	50.9%	146	39.2%		
	Total	606	100%	234	100%	372	100%		
**Stage**	0–II	254	41.8%	80	34%	174	46.8%	9.6	**0.002**
	III–IV	353	58.2%	155	66%	198	53.2%		
	Total	607	100%	235	100%	372	100%		
**Grade**	G1	191	32.2%	50	21.8%	141	38.7%	22.2	**<0.001**
	G2	263	44.4%	108	47.2%	155	42.6%		
	G3	139	23.4%	71	31%	68	18.7%		
	Total	593	100%	229	100%	364	100%		
**p16**	Negative	498	83%	192	83.5%	306	82.7%	0.006	0.806
	Positive	102	17%	38	16.5%	64	17.3%		
	Total	600	100%	230	100%	370	100%		

a TSPO staining intensity scores of 0–1 were considered low and scores 2–3 as high.

Statistical significance was calculated using the chi-square test. p-values < 0.05 were considered statistically significant (shown in bold).

### 3.2 Association of TSPO expression with clinicopathological features

**Figure 1:**
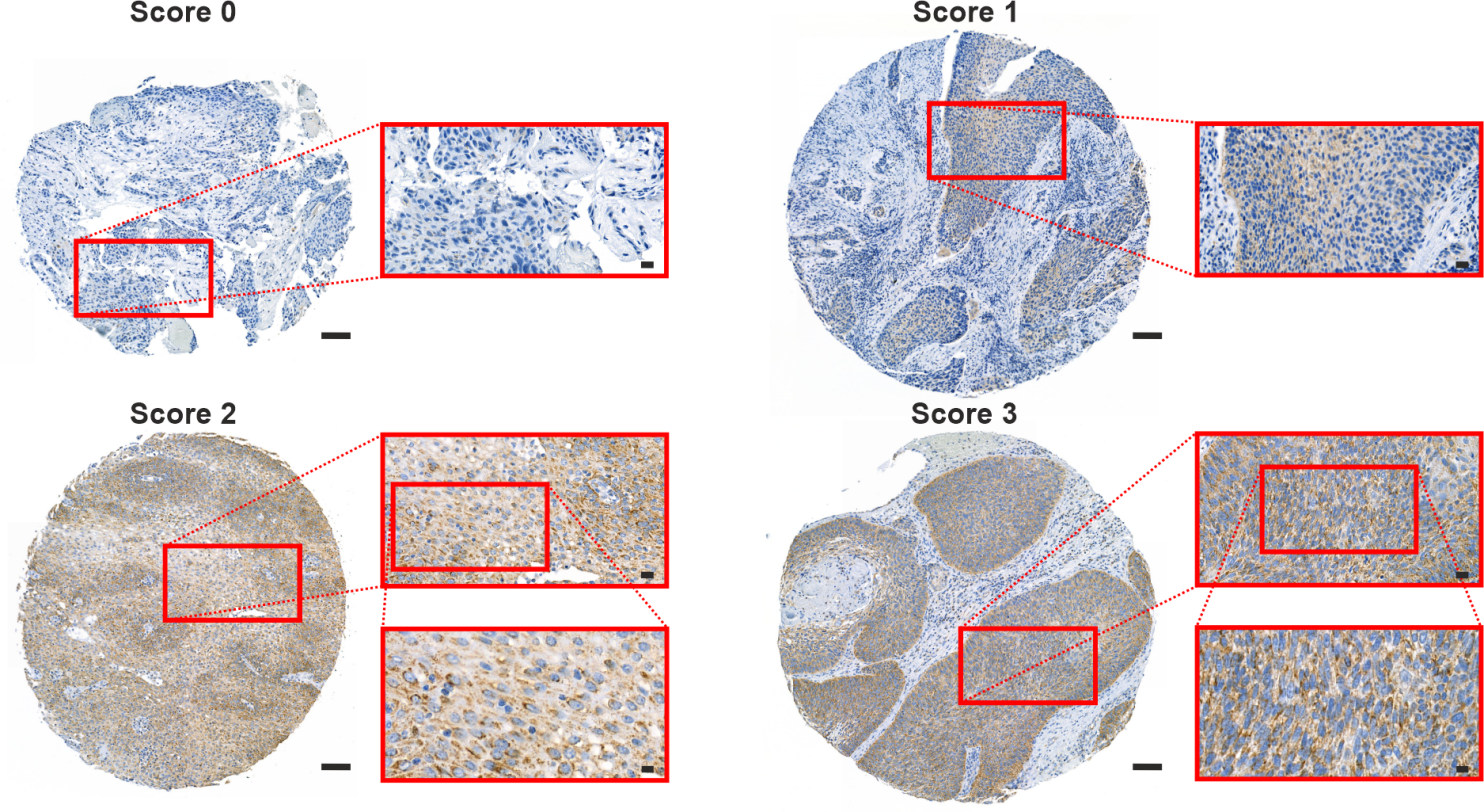
TSPO expression pattern in the HNSCC TMA cohort. Representative images of grade 2 HNSCC sections stained against TSPO. Sections were scored (0–3) according to the staining intensity. Scale bars: 100 µm (whole section), 20 µm (first inset), and 10 µm (second inset).

Representative TMA images of TSPO staining intensities according to scoring (0–3) are shown in **Figure 1**. The staining was mostly cytoplasmic and in line with the known localization of TSPO in mitochondrial membranes. The association of low or high TSPO expression with clinicopathological features is presented in **Table 1**. A trend (p = 0.088) towards higher tumor TSPO expression was detected in patients aged ≥ 65 years (based on median age) compared to younger ones. No significant differences were seen in TSPO expression between males and females or between the primary tumor locations.

Primary tumors with low T classification or no lymph node metastases had significantly (p = 0.015 and p = 0.005, respectively) higher TSPO expression compared to tumors classified as T3–T4 or N+. Similarly, low-staged (0–II) tumors had significantly (p = 0.002) higher TSPO expression compared to high-staged (III–IV) tumors. A significant (p < 0.001) association was found between higher tumor TSPO expression and lower tumor grade. In contrast, no association was found between tumor TSPO and p16 expression.

### 3.3 Survival analyses in clinicopathological subgroups

Univariate and multivariable analyses were performed to study the effect of clinicopathological subgroups or TSPO expression on 5-year survival (**Table 2**). According to univariate analyses, older patients had worse OS and DSS compared to younger patients (p < 0.001 and p = 0.031, respectively); no difference was seen in DFS. Likewise, higher T status, presence of lymph node metastases, and overall stage associated with worse OS, DSS, and DFS (all p < 0.001). Moreover, patients with high-grade tumors (G3) had worse OS (p = 0.005) and DSS (p < 0.001) compared to patients with lower-grade (G1) tumors. No difference was seen in DFS. Patients with G2 tumors had worse DSS (p = 0.011) compared to patients with G1 tumors, but no difference was seen in OS and DFS. As expected, patients with p16-negative tumors had worse survival compared to those with p16-positive tumors (all p ≤ 0.018, see **Table 2**). Interestingly, low tumor TSPO expression associated with worse OS (p = 0.001) and DSS (p < 0.001). A trend towards worse DFS was also seen in patients with lower TSPO expression.

**Table 2:** Univariate and multivariable survival analyses of TSPO expression or clinicopathological subgroups in the HNSCC TMA cohort.

		Univariate						Multivariable					
		OS		DSS		DFS		OS		DSS		DFS	
		HR (95% CI)	p-value	HR (95% CI)	p-value	HR (95% CI)	p-value	HR (95% CI)	p-value	HR (95% CI)	p-value	HR (95% CI)	p-value
**Age** ^a^	<65	1	**-**	1	*-*	1	-	1	**-**	1	**-**	-	*-*
**(y)**	≥65	1.511 (1.193–1.915)	**<0.001**	1.368 (1.027–1.822)	**0.031**	1.241 (0.921–1.671)	0.156	1.677 (1.300–2.163)	**<0.001**	1.578 (1.161–2.143)	**0.004**	-	**-**
**T**	T1–T2	1	**-**	1	*-*	1	-	1	**-**	1	**-**	1	*-*
	T3–T4	3.484 (2.739–4.430)	**<0.001**	4.454 (3.294–6.023)	**<0.001**	2.392 (1.768–3.237)	**<0.001**	2.840 (2.185–3.693)	**<0.001**	3.134 (2.264–4.340)	**<0.001**	2.032 (1.475–2.800)	**<0.001**
**N**	N0	1	-	1	*-*	1	-	1	-	1	-	1	*-*
	N+	1.816 (1.437–2.295)	**<0.001**	2.592 (1.930–3.482)	**<0.001**	1.659 (1.229–2.239)	**<0.001**	1.504 (1.147–1.973)	**0.003**	2.040 (1.456–2.859)	**<0.001**	1.680 (1.211–2.331)	**0.002**
**Stage** ^b^	0–II	1	-	1	*-*	1	-	-	-	-	-	-	*-*
	III–IV	2.613 (2.009–3.400)	**<0.001**	4.130 (2.861–5.962)	**<0.001**	1.884 (1.375–2.581)	**<0.001**	-	**-**	-	**-**	-	**-**
**Grade**	G1	1	-	1	-	1	-	1	-	1	-	-	-
	G2	1.231 (0.924–1.640)	0.156	1.614 (1.117–2.330)	**0.011**	1.099 (0.779–1.551)	0.590	1.066 (0.789–1.440)	0.677	1.244 (0.847–1.826)	0.266	-	**-**
	G3	1.577 (1.149–2.166)	**0.005**	2.020 (1.352–3.019)	**<0.001**	1.052 (0.695–1.594)	0.809	1.520 (1.055–2.190)	**0.024**	1.573 (0.999–2.477)	**0.050**	-	**-**
**p16**	Negative	1	-	1	-	1	-	1	-	1	-	1	-
	Positive	0.568 (0.398–0.809)	**0.002**	0.597 (0.389–0.915)	**0.018**	0.451 (0.276–0.734)	**0.001**	0.518 (0.349–0.767)	**0.001**	0.511 (0.318–0.820)	**0.005**	0.394 (0.238–0.651)	**<0.001**
**TSPO**	Low	1	-	1	-	1	-	1	-	1	-	1	-
	High	0.678 (0.537–0.857)	**0.001**	0.569 (0.429–0.756)	**<0.001**	0.769 (0.569–1.041)	0.089	0.803 (0.623–1.035)	0.090	0.713 (0.524–0.968)	**0.030**	0.941 (0.686–1.290)	0.706

a Age was excluded from DFS multivariable analyses because of its non-significant effect in the univariate analysis.

b The overall stage was excluded from the multivariable analyses because of its direct association with T and N status.

Statistical significance was calculated using Cox’s proportional hazard model. p-values < 0.05 were considered statistically significant (shown in bold).

“-” symbol refers to not applicable.

Multivariable analyses were performed to study independent prognostic factors in the TMA cohort. Age remained a prognostic factor for OS (p < 0.001) and DSS (p = 0.004). Both T and N status, as well as p16 status, were significant prognostic factors for OS, DSS, and DFS (all p ≤ 0.005, see **Table 2**). In addition, high grade (G3) status remained as a prognostic factor for OS (p = 0.024) and DSS (p = 0.050). TSPO was an independent prognostic factor for DSS (p = 0.030), but not for OS and DFS.

### 3.4 Prognostic impact of TSPO in clinicopathological subgroups

Five-year survival rates were analyzed for TSPO expression according to clinicopathological features (**Supplementary Table S4**). A prognostic value of TSPO was found for tumors with lower (all p ≤ 0.045, see **Supplementary Table S4**) but not higher T classification, showing worse survival when TSPO expression was low. Lower TSPO expression in both N0- and N+-classified tumors indicated worse OS (p = 0.058 and p = 0.044, respectively) and DSS (p = 0.018 and p = 0.022, respectively). Similarly, low TSPO levels were associated with worse DSS (p = 0.013 in stage 0–II and p = 0.042 in stage III–IV) in all tumor stages. A trend towards significantly (p = 0.063) worse DSS was found for patients with low TSPO-expressing tumors of grade G1, whereas grade G2 tumors with low TSPO expression associated with worse OS (p = 0.011) and DSS (p = 0.009). Such association was not found for grade G3 tumors.

### 3.5 Site-specific survival analyses

**Figure 2:**
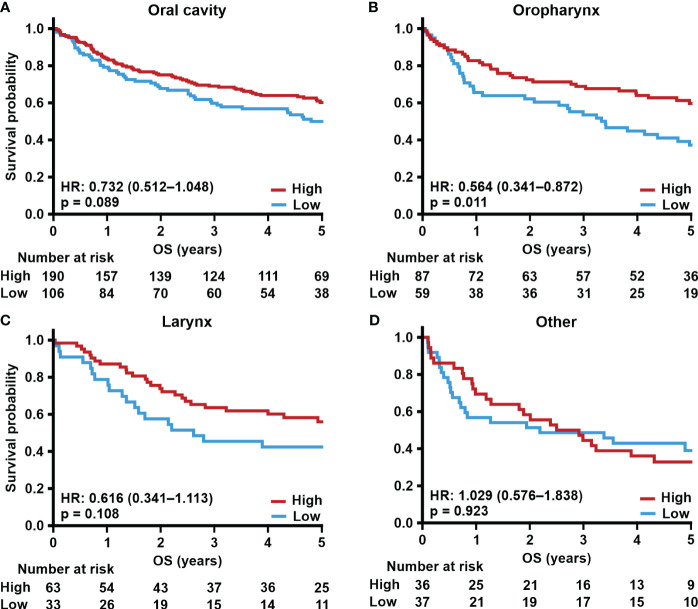
TSPO expression and site-specific overall survival. Prognostic trends with HR (95% CI) for 5-year OS in **(A)** oral cavity, **(B)** oropharynx, **(C)** larynx, and **(D)** other primary tumor sites divided into low and high (staining intensity scores of 0–1 and 2–3, respectively) TSPO tumor expression. Statistical significance was calculated using Cox’s proportional hazard model. p-values < 0.05 were considered statistically significant.

**Figure 3:**
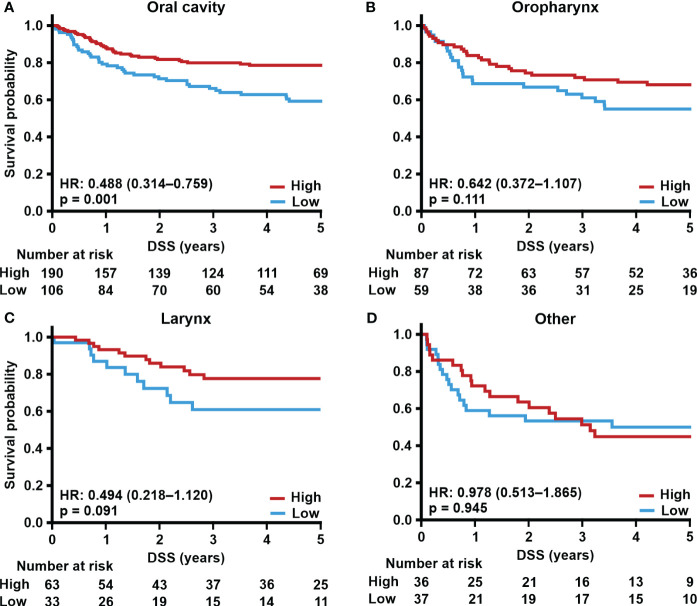
TSPO expression and site-specific disease-specific survival. Prognostic trends with HR (95% CI) for 5-year DSS in **(A)** oral cavity, **(B)** oropharynx, **(C)** larynx, and **(D)** other primary tumor sites divided into low and high (staining intensity scores of 0–1 and 2–3, respectively) TSPO tumor expression. Statistical significance was calculated using Cox’s proportional hazard model. p-values < 0.05 were considered statistically significant.

**Figure 4:**
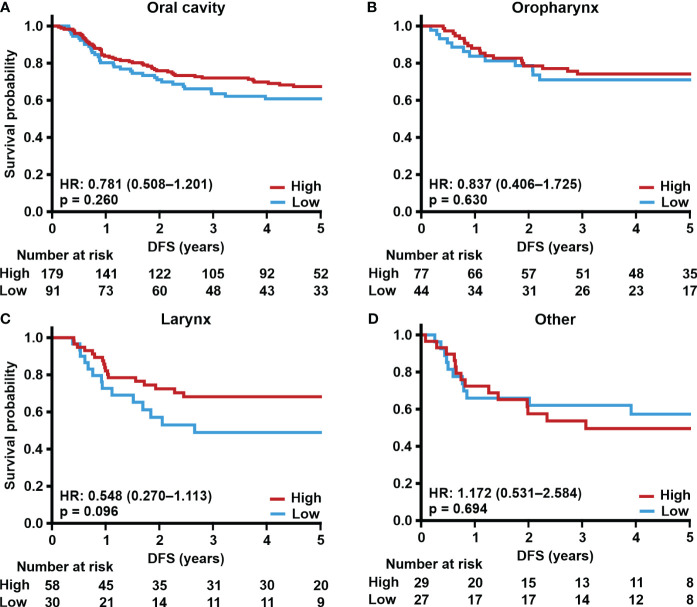
TSPO expression and site-specific disease-free survival. Prognostic trends with HR (95% CI) for 5-year DFS in **(A)** oral cavity, **(B)** oropharynx, **(C)** larynx, and **(D)** other primary tumor sites divided into low and high (staining intensity scores of 0–1 and 2–3, respectively) TSPO tumor expression. Statistical significance was calculated using Cox’s proportional hazard model. p-values < 0.05 were considered statistically significant.

Next, we correlated TSPO expression in primary tumors from different locations with survival. Tumors with low TSPO expression originating from the oral cavity (**Figure 2A**) showed a trend (p = 0.089) towards worse OS. In oropharyngeal (**Figure 2B**) cancer OS showed a significant (p = 0.011) association between lower TSPO expression and worse survival. No difference in OS between low and high TSPO expression was seen in larynx (**Figure 2C**) and tumors originating from ‘Other’ sites (**Figure 2D**). An association between worse DSS and low TSPO expression (p = 0.001) was found in tumors located in the oral cavity (**Figure 3A**), and a trend (p = 0.091) was seen in laryngeal cancer (**Figure 3C**). No differences in DSS were seen in oropharyngeal cancer (**Figure 3B**) and ‘Other’ tumors (**Figure 3D**). Only laryngeal cancer (**Figure 4B**) showed a trend (p = 0.096) towards worse DFS (**Figure 4**) in low TSPO expressing tumors.

### 3.6 Effect of p16 and TSPO protein expression on survival

We also evaluated the prognostic role of TSPO expression in the whole TMA cohort with tumor p16 status (**Supplementary Figure S2**). Regardless of p16 status, lower TSPO expression associated with worse OS and DSS (p = 0.015–0.001, see **Supplementary Figure S2**). However, no association was found between DFS and TSPO expression in p16-negative tumors, whereas higher TSPO expression was associated with better DFS in p16-positive tumors (p = 0.028).

**Figure 5:**
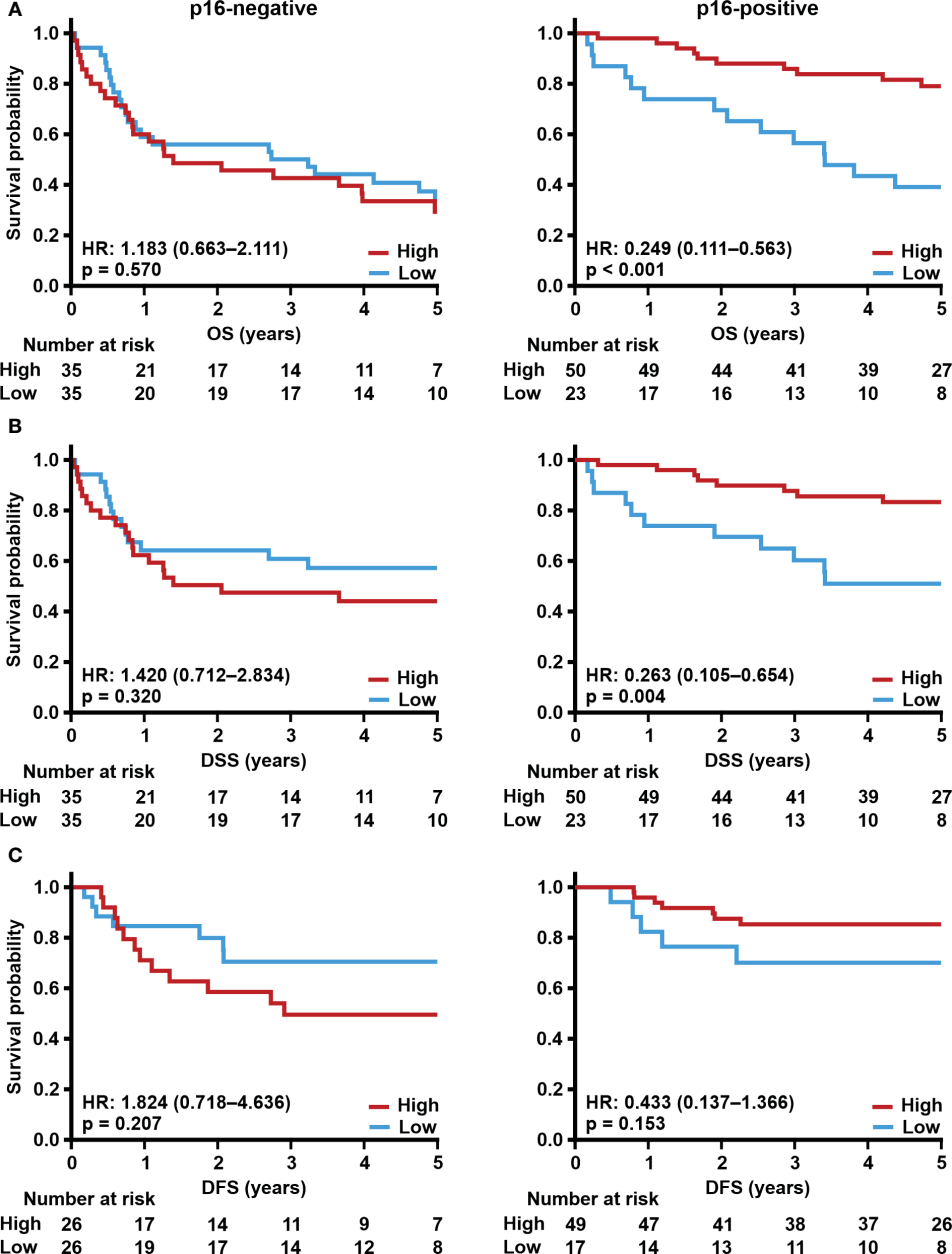
Effect of p16 and TSPO expression on survival. Prognostic trends with HR (95% CI) for 5-year **(A)** OS, **(B)** DSS, and **(C)** DFS in oropharyngeal cancer according to low (staining intensity scores 0–1) and high (staining intensity scores 2–3) TSPO tumor expression in patients with p16-negative or p16-positive tumors. Statistical significance was calculated using Cox’s proportional hazard model. p-values < 0.05 were considered statistically significant.

As p16-positive tumors are most often located in the oropharyngeal area, we separately analyzed the prognostic value of TSPO in this subgroup of patients. No association between TSPO expression and survival (OS, DSS, and DFS) in p16-negative oropharyngeal cancer was seen (**Figures 5A–C**). However, in p16-positive tumors, lower tumor TSPO expression associated with worse OS (p < 0.001, **Figure 5A**) and DSS (p = 0.004, **Figure 5B**). No difference was seen in DFS (**Figure 5C**).

### 3.7 Prognostic significance of *TSPO* in the TCGA HNSCC cohort

**Figure 6:**
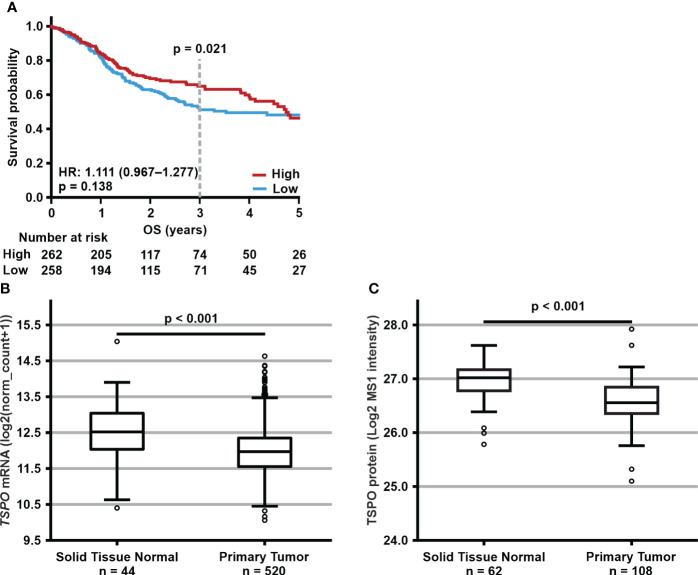
Expression levels and prognostic significance of TSPO expression in the publicly available HNSCC cohorts. **(A)** Prognostic trend with HR (95% CI) for 5-year OS according to low (below median) and high (equal to or above median) *TSPO* expression in all TCGA HNSCC cohort patients. p-value for 3-year OS is shown above the dotted line. Statistical significance for survival analyses was calculated using Cox’s proportional hazard model. **(B)** *TSPO* mRNA and **(C)** TSPO protein expression in normal tissues and primary tumors in TCGA and CPTAC HNSCC cohorts, respectively. Data are shown as minimum, first quartile, median, third quartile, and maximum. Dots represent outliers. Independent samples t-test was used to analyze the difference in *TSPO* expression between normal tissue and primary tumor.

Next, we used the TCGA HNSCC cohort to determine whether our prognostic findings regarding TSPO expression could be reproduced at the mRNA level. Our *in silico* analyses indeed showed that low *TSPO* expression tended to associate with worse OS (**Figure 6A**). Statistical significance was seen at 3-years (p = 0.021) but diminished towards the 5-year end time point. No associations were seen for DSS, disease-free interval, or progression-free interval (**Supplementary Figure S3**). Both *TSPO* mRNA and TSPO protein expression were higher in healthy corresponding tissue compared to tumor (p < 0.001) (**Figures 6B, C**), showing significantly lower *TSPO* expression in tumors originating from the oral cavity (p = 0.001), larynx (p = 0.002), tonsils (p = 0.031), and tongue (p < 0.001) (**Supplementary Figure S3A**).

Non-malignant tissue also expressed higher *TSPO* levels than carcinomas regardless of N or T classification or overall staging (all p < 0.001, see **Supplementary Figures S4B–D**). In addition, normal tissue had significantly (p < 0.001) higher *TSPO* expression compared to grade G2 and G3 tumors (**Supplementary Figure S4E**). *TSPO* expression was also downregulated in tumors with more aggressive histological characteristics as G1 tumors had significantly higher *TSPO* expression compared to G2 (p = 0.036) tumors. No difference was seen in *TSPO* expression between p16-negative and p16-positive tumors (**Supplementary Figure S4F**). We observed no mutations or copy number alterations targeting *TSPO* in the TCGA dataset.

Site-specific survival analyses revealed a significantly worse 5-year (p = 0.013) and 3-year (p = 0.004) OS and 3-year progression-free interval (p = 0.043) in patients with laryngeal cancer and low tumor *TSPO* expression, whereas no other site-specific associations were found (**Supplementary Figure S5**). Because of the small number of patients, it was not possible to perform statistical analyses in subgroups of oropharyngeal and hypopharyngeal tumors.

### 3.8 *In silico* analyses reveal a potential functional role of TSPO in HNSCC

**Figure 7:**
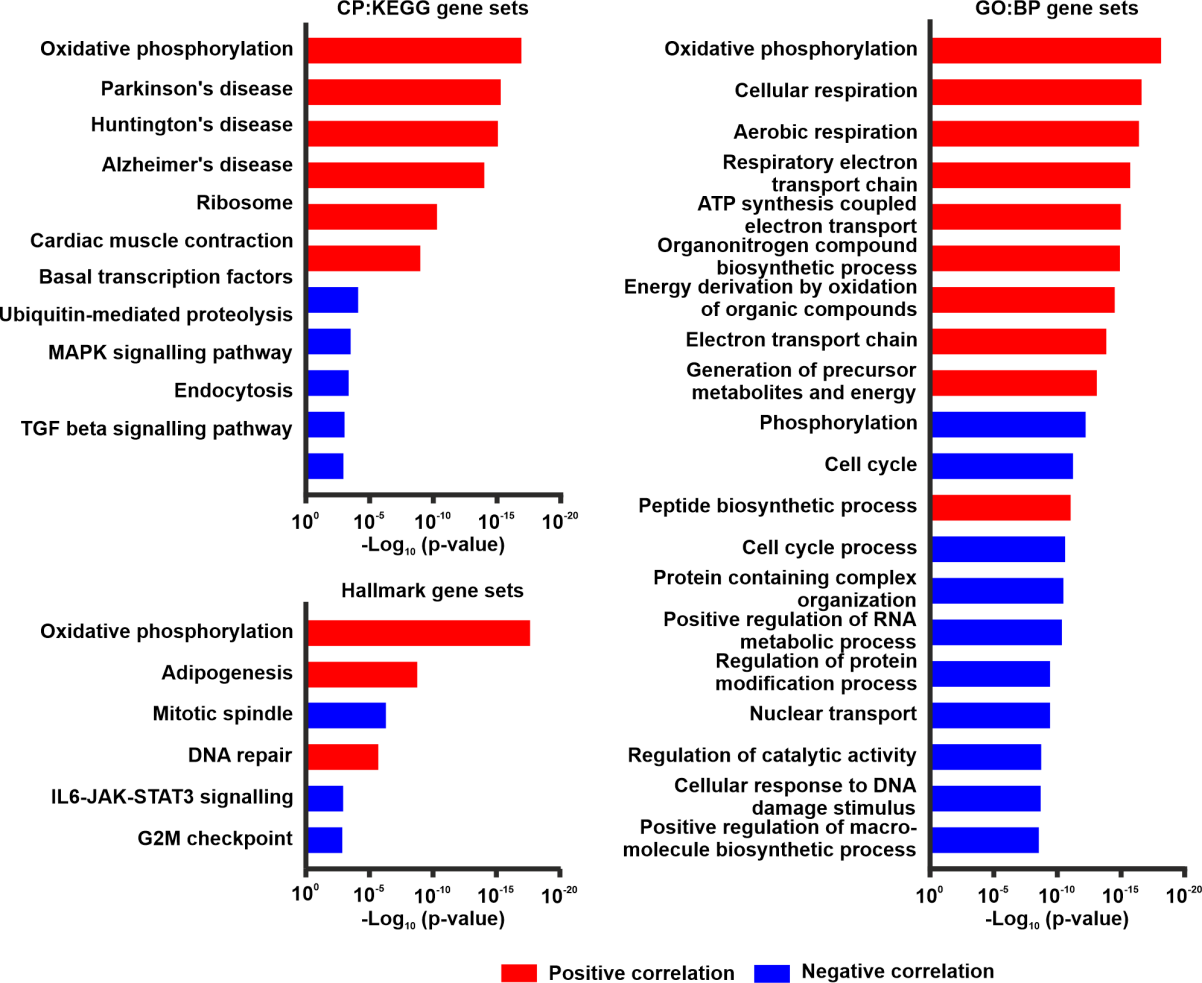
Pathway overlap analyses in the TCGA HNSCC cohort. Overlap analysis for top 100 genes expressed either positively (red) or negatively (blue) with *TSPO*. The top 10 results for both positive and negative correlations are shown for the CP : KEGG, Hallmark, and GO : BP gene set collections. p-values calculated by hypergeometric distribution for pathway gene overlap analyses were acquired from MSigDB.

To decipher which biological processes and pathways TSPO is involved in HNSCC, we performed pathway gene overlap analysis of genes strongly co-expressed with *TSPO*. The top 10 gene set overlaps for each gene set collection (CP : KEGG, Hallmark and GO : BP) of genes positively or negatively correlated with *TSPO* are shown in **Figure 7**. Oxidative phosphorylation had the highest statistical significance in all three gene set collections for genes expressed positively with *TSPO*. In addition, other pathways related to aerobic respiration had gene overlaps in both the GO : BP and CP : KEGG gene sets. Pathway gene overlaps for DNA damage response were found in the Hallmark gene sets for DNA repair in positively expressed genes and in the GO : BP gene sets for cellular response to DNA damage stimulus in negatively expressed genes. Furthermore, pathway gene overlap for adipogenesis was found in the Hallmark gene sets. Other significant findings were found for diseases related to the central nervous system and pathways related to cell cycle regulation. A list of gene names and gene set overlaps for genes positively and negatively expressed with *TSPO* are shown in **Supplementary Tables S5**, **S6**, respectively.

**Figure 8:**
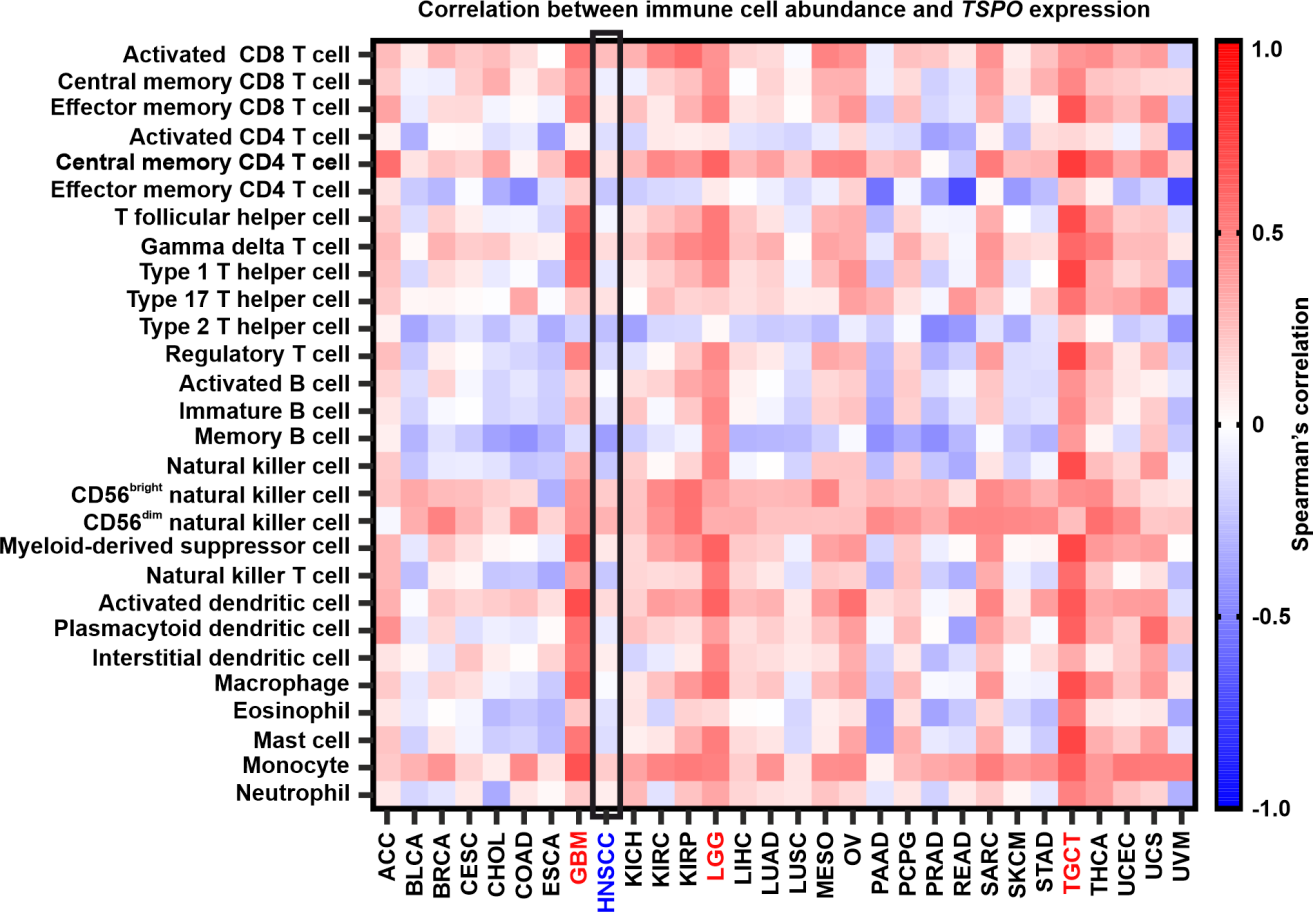
Correlation between *TSPO* expression and immune cell abundance in different cancers. HNSCC (blue) is highlighted inside a black box. GBM, LGG, and TGCT (all in red) showed the highest positive correlations. Spearman’s correlations between immune cell abundance and *TSPO* expression were acquired from TISIDB. GBM, glioblastoma multiforme; LGG, brain lower grade glioma; TGCT, testicular germ cell tumors. The remaining cancer abbreviations are found in **Supplementary Table S7**.

Due to the previously suggested immunomodulatory role of TSPO in neuroinflammation (23), we studied the correlation of *TSPO* expression with the abundance of immune cell types in HNSCC and other cancer types (**Figure 8**). While some correlation analyses reached statistical significance in HNSCC, the strength of the associations was substantially less pronounced compared to many other cancer types. In HNSCC (**Supplementary Figure S6**), the most prominent positive correlations with *TSPO* expression were observed with activated CD8 T cells, CD56^dim^, and CD56^bright^ natural killer cells, whereas the most significant negative associations were observed with memory B cells, type 2 T helper cells, and effector memory CD4 T cells. In addition, *TSPO* expression was positively correlated with most immune cell types in both glioblastoma (**Supplementary Figure S7**) and lower-grade glioma (**Supplementary Figure S8**), except for negative correlations observed with type 2 T helper cells and memory B cells. *TSPO* expression was also correlated with immune cells in other cancers, displaying the strongest association with testicular germ cell tumors (**Supplementary Figure S9**).

## 4 Discussion

Improved biomarkers are urgently needed in HNSCC to better guide clinical decision making. TSPO has previously been reported to be overexpressed as well as associated with poor survival in several cancer types. We used a large population-based HNSCC TMA cohort and publicly available dataset to determine whether TSPO expression levels associated with any of several clinicopathological features and survival.

Surprisingly, in contrast to most previous research, we found that lower TSPO expression associated with higher tumor grade, staging, and worse survival rates. Our results clearly demonstrate that TSPO expression is a significant prognostic factor for OS and DSS in HNSCC. TSPO also remained an independent predictor of DSS. *In silico* results also supported this finding showing better patient 3-year OS with high tumor *TSPO* expression. However, our *in silico* analyses found no difference in DSS and DFS, in contrast to the TMA findings. Moreover, TSPO expression was not significantly different between primary tumors originating from distinct anatomical sites, but a trend towards a positive association between higher tumor TSPO expression and patient age was seen. We also found that *TSPO* mRNA and TSPO protein expression were downregulated in HNSCC compared to normal comparable tissue in dataset analyses. Previously, both *TSPO* over- and underexpression have been reported in different types of cancers compared to their healthy counterpart tissues (20). The regulators of TSPO expression in HNSCC remain to be studied, but likely both transcriptional and post-translational regulation are involved. We observed no genetic aberrations targeting *TSPO* in TCGA data.

HNSCC is a heterogeneous disease including tumors arising from different anatomical sites with distinct histological types and HPV status. Previously, TSPO expression in HNSCC had only been studied in a small cohort of patients with carcinomas of the oral cavity (41). This study by Nagler et al. reported a strong association between higher TSPO expression and patient mortality. After dividing our cohort into site-specific subgroups, we assessed the prognostic value of TSPO in all evaluated tumor sites. In contrast to the findings by Nagler et al., we observed that higher TSPO expression associated with better survival in carcinomas not only of the oral cavity, but also the larynx and oropharynx. Our population-based cohort consists of TMAs from over 600 patients, and thus more likely excludes the possibility of biased findings. However, the use of different antibodies may cause discrepancies between different studies. Our *in silico* results showed a clear association between lower *TSPO* expression and survival in laryngeal cancer, in line with the TMA findings. Such association was lacking in other subsites, possibly due to the small number of patients in some groups.

We also evaluated the prognostic value of TSPO expression in p16-positive and -negative tumors. A significant prognostic value for TSPO was found regardless of p16 status when all patients with HNSCC were evaluated. However, in oropharyngeal cancer, which is the most common site for HPV infection, survival was significantly worse in patients with p16-positive tumors and low TSPO expression. No differences in survival were seen in patients with p16-negative oropharyngeal cancer irrespective of TSPO expression level. Unfortunately, it was not possible to reliably study the effect of *TSPO* on survival according to p16 status in the TCGA cohort due to the limited number of patients.

The functional role of TSPO in HNSCC is not well understood, but our *in silico* findings support previous studies reporting that TSPO is implicated in the regulation of oxidative phosphorylation and cellular respiration processes (27, 57–59). Previous physiological and pathological findings also support the role of TSPO in cellular metabolism (60, 61). Moreover, a recent study showed that high expression of genes involved in oxidative phosphorylation is associated with improved survival in HNSCC (62). However, a contradictive finding suggesting that increased oxidative phosphorylation in general is associated with a worse outcome in HNSCC has also been published (63).

HPV-positive tumors rely on oxidative phosphorylation, whereas aerobic glycolysis is activated in HPV-negative tumors (64–66). In addition, dysregulation of oxidative phosphorylation may affect treatment failure in recurrent HPV-induced diseases (67). Our results provide a possible connection between TSPO and p16 in oropharyngeal cancer, which we hypothesize to be related to mitochondrial functionalities, such as oxidative phosphorylation. We have previously shown that uptake of the TSPO-PET tracer, [^18^F]F-DPA, increases after irradiation in HNSCC cells and tumor xenografts (45). This increased tracer uptake, which was shown to be TSPO-specific, was not caused by higher TSPO expression, hence indicating changes in TSPO functionality after irradiation. Further studies are warranted to clarify the relationship between TSPO and p16 as well as determine whether TSPO regulates oxidative phosphorylation during radiotherapy. PET imaging of TSPO is intensively used to image neuroinflammation in a diverse range of neurodegenerative conditions (23) and malignant brain gliomas (68), as TSPO is overexpressed in activated microglia and macrophages (69). In our previous preclinical PET study with HNSCC xenografts, we did not find a clear connection between [^18^F]F-DPA uptake and macrophages (45). Therefore, we characterized the TSPO-associated immune landscape in HNSCC *in silico*. In addition, immune checkpoint blockade has emerged as an important effective therapeutic option in HNSCC. Overall, the data indicate that while TSPO may play a role in the immune landscape of some cancers, its potential immunomodulatory role may be less important in the context of HNSCC. Our findings with gliomas are in line with previous studies proposing an immunomodulatory role for TSPO in the central nervous system (27). The highest positive correlations with *TSPO* were found with CD56^dim^ natural killer cells and CD8 T cells, which have been previously associated with better survival in HNSCC and are in line with our data (70).

Main study limitations were the low number of patients for some primary tumor sites and the retrospective study design. Several statistical comparisons were performed and the possibility of false positive findings cannot be ruled out. Nevertheless, multivariable analyses were performed to reduce the probability of biased findings and we consider our statistical approach robust with the available data. Our TMA study cohort was large, including all HNSCC patients diagnosed and treated in southwest Finland within an 11-year period. No risk of inclusion bias due to socioeconomic or health-insurance status occurred as all patients are referred to tertiary referral centers and treated according to the national treatment guidelines. Only one publicly accessible HNSCC patient cohort (TCGA) was available for comprehensive *in silico* analyses and the low sample number in some primary tumor sites of this dataset further limited our analyses.

In conclusion, our results consistently show that TSPO is a potential independent prognostic biomarker in HNSCC. Low TSPO expression is robustly correlated with advanced disease stage and worse survival. We hypothesize that decreased TSPO functionality reflects reduced cellular respiration and oxidative metabolism capacity, leading to treatment resistance and poor survival. Further studies are, however, warranted to clarify the regulative role of TSPO in HNSCC and whether the association between p16 and TSPO is of clinical significance.

## Data availability statement

The datasets presented in this study can be found in online repositories. The names of the repository/repositories and accession number(s) can be found in the article.

## Ethics statement

The studies involving humans were approved by the institutional review board of the Finnish national authority for medicolegal affairs, regional ethics committee of University of Turku, and Auria Biobank scientific board. The studies were conducted in accordance with the local legislation and institutional requirements. The human samples used in this study were acquired from Auria Biobank (Wellbeing services county of Southwest Finland). Written informed consent for participation was not required from the participants or the participants’ legal guardians/next of kin in accordance with the national legislation and institutional requirements.

## Author contributions

ST: Conceptualization, Data curation, Formal analysis, Funding acquisition, Investigation, Methodology, Writing – original draft, Writing – review & editing. LN: Data curation, Formal analysis, Funding acquisition, Investigation, Methodology, Writing – review & editing. AK: Data curation, Formal analysis, Funding acquisition, Investigation, Methodology, Writing – original draft, Writing – review & editing. JR: Data curation, Writing – review & editing. TH: Data curation, Writing – review & editing. IL: Data curation, Writing – review & editing. HM: Writing – review & editing. HI: Writing – review & editing. EL: Data curation, Formal analysis, Writing – original draft, Writing – review & editing. SV: Data curation, Writing – review & editing. MS: Conceptualization, Project administration, Writing – original draft, Writing – review & editing. TG: Conceptualization, Funding acquisition, Project administration, Writing – original draft, Writing – review & editing.
