# Supplementary material for: TSPO is a potential independent prognostic factor associated with cellular respiration and p16 in head and neck squamous cell carcinoma
Source: Front Oncol. 2023 Dec 14;13:1298333. doi: 10.3389/fonc.2023.1298333 (PMC10755888; doi:10.3389/fonc.2023.1298333)
Supplement: Supplementary file 1 [file DataSheet_1.pdf]

TSPO is a potential independent prognostic factor associated with cellular respiration and p16 in head and neck squamous cell carcinoma

Sanni Tuominen[1,2,3], Linda Nissi[4], Antti Kukkula[2], Johannes Routila[5], Teemu Huusko[5], Ilmo Leivo[6], Heikki Minn[4], Heikki Irjala[5], Eliisa Löyttyniemi[7], Sami Ventelä[5], Maria Sundvall[2,4] and Tove J. Grönroos[1,3,4]

[1]Preclinical Imaging Laboratory, Turku PET Centre, University of Turku, Turku, Finland

[2]Cancer Research Unit, Institute of Biomedicine, and FICAN West Cancer Research Laboratory, Turku University Hospital and University of Turku, Turku, Finland

[3]Medicity Research Laboratory, Faculty of Medicine, University of Turku, Turku, Finland

[4]Department of Clinical Oncology, Turku University Hospital and University of Turku, Turku, Finland

[5]Department of Otorhinolaryngology – Head and Neck Surgery, Turku University Hospital and University of Turku, Turku, Finland

[6]Department of Pathology, Turku University Hospital and University of Turku, Turku, Finland

[7]Department of Biostatistics, Turku University Hospital and University of Turku, Turku, Finland

Supplementary Table S1. The number of patients with HNSCC in subgroups from which *TSPO* expression was available and used in the *in silico* analyses from the TCGA cohort.

		No. of patients	*TSPO* mRNA level (log2(norm_value+1))	Data version
**Sample type**	Solid tissue normal	74	44	2019-12-06
	Primary	528	520	
	Total	609	566	
**Site**	Alveoral ridge	18	18	2019-12-06
	Base of tongue	27	27	
	Buccal mucosa	23	22	
	Floor of mouth	63	62	
	Hard palate	7	7	
	Hypopharynx	10	10	
	Larynx	117	116	
	Lip	3	3	
	Oral cavity	73	73	
	Oral tongue	133	130	
	Oropharynx	9	9	
	Tonsil	45	43	
	Total	528	520	
	Missing	0	0	
**T**	T1	37	35	2019-12-06
	T2	152	151	
	T3	139	135	
	T4	25	25	
	T4a	156	155	
	T4b	3	3	
	TX	12	12	
	Total	524	516	
	Missing	4	4	
**N**	N0	246	244	2019-12-06
	N1	85	83	
	N2	19	19	
	N2a	17	17	
	N2b	85	81	
	N2c	45	45	
	N3	9	9	
	NX	18	18	
	Total	524	516	
	Missing	4	4	
**Stage**	I	21	20	2019-12-06
	II	99	98	
	III	107	105	
	IVA	269	265	
	IVB	11	11	
	IVC	7	7	
	Total	514	506	
	Missing	14	14	
**Grade**	G1	63	62	2019-12-06
	G2	311	304	
	G3	125	125	
	G4	7	7	
	GX	18	18	
	Total	524	516	
	Missing	4	4	
**p16**	Negative	74	73	2019-12-06
	Positive	41	38	
	Total	115	111	
	Missing	413	409	

Supplementary Table S2. Top 100 genes with the highest positive expression with *TSPO*.

Correlated gene	Cytoband	Spearman's correlation	p-Value	q-Value
MPST	22q12.3	0.661	1.31E-62	2.64E-58
NOL12	22q13.1	0.604	6.07E-50	6.08E-46
UQCR10	22q12.2	0.597	1.68E-48	1.12E-44
TRABD	22q13.33	0.588	1.02E-46	3.41E-43
RBX1	22q13.2	0.587	1.29E-46	3.71E-43
SMDT1	22q13.2	0.569	3.91E-43	9.81E-40
MCAT	22q13.2	0.567	6.36E-43	1.42E-39
TXN2	22q12.3	0.565	1.97E-42	3.58E-39
NDUFB7	19p13.12	0.562	4.83E-42	6.92E-39
COMTD1	10q22.2	0.559	1.61E-41	1.61E-38
PMVK	1q21.3	0.553	1.75E-40	1.52E-37
PGLS	19p13.11	0.553	1.86E-40	1.55E-37
MIF	22q11.23	0.551	4.66E-40	3.60E-37
NDUFA6	22q13.2	0.548	1.26E-39	9.40E-37
STAP2	19p13.3	0.546	2.63E-39	1.82E-36
RPS19BP1	22q13.1	0.545	4.50E-39	2.79E-36
SNU13	22q13.2	0.545	4.58E-39	2.79E-36
SELENOO	22q13.33	0.543	9.53E-39	5.46E-36
SCO2	22q13.33	0.543	1.08E-38	5.99E-36
MVB12A	19p13.11	0.539	4.05E-38	2.20E-35
GGA1	22q13.1	0.535	1.62E-37	8.35E-35
FBXL15	10q24.32	0.534	2.17E-37	1.09E-34
HSPB1	7q11.23	0.531	6.67E-37	3.04E-34
PMM1	22q13.2	0.531	7.31E-37	3.26E-34
GIPC1	19p13.12	0.531	8.21E-37	3.58E-34
NDUFA13	19p13.11	0.524	1.04E-35	3.78E-33
COPE	19p13.11	0.522	1.78E-35	5.93E-33
COX4I1	16q24.1	0.520	3.16E-35	9.74E-33
RPS15	19p13.3	0.519	4.47E-35	1.32E-32
WDR83OS	19p13.13	0.519	4.93E-35	1.42E-32
ZNF524	19q13.42	0.519	5.35E-35	1.51E-32
VAMP8	2p11.2	0.518	7.03E-35	1.93E-32
AURKAIP1	1p36.33	0.518	8.48E-35	2.24E-32
ATP5F1D	19p13.3	0.517	9.23E-35	2.37E-32
ZMAT5	22q12.2	0.517	1.06E-34	2.65E-32
POLR2F	22q13.1	0.516	1.38E-34	3.37E-32
COX8A	11q13.1	0.516	1.53E-34	3.66E-32
PICK1	22q13.1	0.514	2.60E-34	5.80E-32
RPS9	19q13.42	0.514	3.19E-34	6.94E-32
TRAPPC5	19p13.2	0.513	4.88E-34	1.04E-31
GUK1	1q42.13	0.512	5.28E-34	1.09E-31
TRADD	16q22.1	0.511	7.21E-34	1.46E-31
CCDC124	19p13.11	0.510	1.06E-33	2.09E-31
TEN1	17q25.1	0.509	1.55E-33	2.94E-31
FAM98C	19q13.2	0.509	1.66E-33	3.12E-31
GADD45GIP1	19p13.13	0.507	3.17E-33	5.73E-31
SGSM3	22q13.1	0.504	7.20E-33	1.27E-30
STUB1	16p13.3	0.504	7.58E-33	1.32E-30
HSPB1P1	9q21.13	0.504	9.22E-33	1.58E-30
ILVBL	19p13.12	0.503	1.25E-32	2.08E-30
TRIR	19p13.13	0.502	1.50E-32	2.44E-30
TBC1D22A	22q13.31	0.500	3.31E-32	4.99E-30
NME3	16p13.3	0.499	3.83E-32	5.69E-30
DDT	22q11.23	0.499	4.19E-32	6.18E-30
PRR34-AS1	22q13.31	0.499	4.40E-32	6.44E-30
COQ4	9q34.11	0.498	5.12E-32	7.38E-30

Supplementary Table S2 (continued). Top 100 genes with the highest positive expression with *TSPO*.

Correlated Gene	Cytoband	Spearman's correlation	p-Value	q-Value
MRPS34	16p13.3	0.498	6.72E-32	9.43E-30
RPL36	19p13.3	0.497	7.33E-32	1.02E-29
ECHS1	10q26.3	0.496	1.12E-31	1.53E-29
NDUFA11	19p13.3	0.495	1.61E-31	2.19E-29
PERM1	1p36.33	0.494	1.98E-31	2.65E-29
EDF1	9q34.3	0.493	2.56E-31	3.36E-29
TMEM134	11q13.2	0.493	3.48E-31	4.47E-29
RPL18A	19p13.11	0.492	4.21E-31	5.28E-29
ARF5	7q32.1	0.491	5.20E-31	6.36E-29
COPS9	2q37.3	0.491	5.30E-31	6.45E-29
UBA52	19p13.1-p12	0.490	6.81E-31	8.04E-29
ATP5MK	10q24.33	0.490	7.71E-31	8.99E-29
NDUFS7	19p13.3	0.490	8.12E-31	9.42E-29
SSNA1	9q34.3	0.489	1.08E-30	1.23E-28
UQCRQ	5q31.1	0.489	1.17E-30	1.32E-28
PYM1	12q13.2	0.488	1.30E-30	1.46E-28
S100A16	1q21.3	0.488	1.51E-30	1.68E-28
JOSD2	19q13.33	0.486	2.60E-30	2.82E-28
ALKBH7	19p13.3	0.486	2.96E-30	3.13E-28
COA3	17q21.2	0.485	3.25E-30	3.41E-28
COX6A1	12q24.31	0.485	3.32E-30	3.47E-28
ABHD17A	19p13.3	0.485	3.65E-30	3.79E-28
VPS28	8q24.3	0.485	3.66E-30	3.79E-28
NDUFA7	19p13.2	0.484	4.97E-30	5.06E-28
RPL37A	2q35	0.484	5.40E-30	5.47E-28
NR2F6	19p13.11	0.483	6.41E-30	6.37E-28
RAB24	5q35.3	0.483	7.64E-30	7.55E-28
RPL34	4q25	0.482	8.64E-30	8.41E-28
TMEM191A	22q11.21	0.482	9.02E-30	8.69E-28
CCDC12	3p21.31	0.481	1.34E-29	1.27E-27
TOMM22	22q13.1	0.480	1.86E-29	1.73E-27
INKA1	3p21.31	0.478	3.10E-29	2.76E-27
MRPL41	9q34.3	0.477	4.76E-29	4.13E-27
TST	22q12.3	0.476	4.99E-29	4.28E-27
RPS19	19q13.2	0.475	7.68E-29	6.44E-27
COX7A2	6q14.1	0.474	9.64E-29	7.83E-27
PRDX5	11q13.1	0.474	9.96E-29	8.02E-27
SNRPD3	22q11.23	0.474	1.13E-28	9.00E-27
UQCR11	19p13.3	0.474	1.13E-28	9.00E-27
NUDT8	11q13.2	0.474	1.18E-28	9.36E-27
UBL5	19p13.2	0.473	1.48E-28	1.16E-26
TRMU	22q13.31	0.473	1.50E-28	1.17E-26
ATF4	22q13.1	0.473	1.52E-28	1.18E-26
YIF1B	19q13.2	0.473	1.59E-28	1.22E-26

Supplementary Table S3. Top 100 genes with the highest negative expression with *TSPO*.

Correlated gene	Cytoband	Spearman's correlation	p-Value	q-Value
ROCK2	2p25.1	-0.597	2.26E-48	1.13E-44
ROCK1	18q11.1	-0.593	1.38E-47	5.54E-44
RIF1	2q23.3	-0.566	1.17E-42	2.35E-39
TRIM44	11p13	-0.564	2.17E-42	3.59E-39
NEU3	11q13.4	-0.564	2.33E-42	3.59E-39
HELZ	17q24.2	-0.561	7.18E-42	8.85E-39
TTBK2	15q15.2	-0.561	7.23E-42	8.85E-39
ZNF281	1q32.1	-0.561	7.50E-42	8.85E-39
ZKSCAN8	6p21	-0.561	9.75E-42	1.09E-38
TAF1L	9p21.1	-0.560	1.43E-41	1.51E-38
MED13	17q23.2	-0.556	7.10E-41	6.78E-38
NF1	17q11.2	-0.555	8.61E-41	7.85E-38
LATS1	6q25.1	-0.552	2.64E-40	2.12E-37
ATRX	Xq21.1	-0.547	2.08E-39	1.49E-36
RGPD4	2q12.3	-0.546	3.40E-39	2.28E-36
PHLPP2	16q22.2	-0.545	4.07E-39	2.63E-36
MAP3K2	2q14.3	-0.544	5.41E-39	3.19E-36
MED1	17q12	-0.538	6.24E-38	3.29E-35
UBR1	15q15.2	-0.534	2.75E-37	1.35E-34
TCF12	15q21.3	-0.533	3.38E-37	1.61E-34
MACF1	1p34.3	-0.532	4.99E-37	2.33E-34
RGPD3	2q12.2	-0.530	1.12E-36	4.77E-34
RAD54L2	3p21.2	-0.529	1.76E-36	7.34E-34
LIMS1	2q12.3	-0.528	2.38E-36	9.72E-34
MIGA1	1p31.1	-0.528	2.55E-36	1.02E-33
CCNT1	12q13.11-q13.12	-0.526	4.15E-36	1.63E-33
ERCC6L2	9q22.32	-0.526	5.17E-36	1.99E-33
ERCC4	16p13.12	-0.524	8.16E-36	3.09E-33
GCC2	2q12.3	-0.524	8.32E-36	3.09E-33
ZNF81	Xp11.23	-0.523	1.48E-35	5.22E-33
ASAP1	8q24.21-q24.22	-0.523	1.48E-35	5.22E-33
PIK3C2A	11p15.1	-0.522	1.57E-35	5.45E-33
ZNF106	15q15.1	-0.522	1.61E-35	5.47E-33
ASXL2	2p23.3	-0.522	1.84E-35	6.05E-33
RBM27	5q32	-0.522	1.92E-35	6.20E-33
GPATCH8	17q21.31	-0.522	2.04E-35	6.51E-33
ADAT1	16q23.1	-0.522	2.13E-35	6.67E-33
SMURF2	17q23.3-q24.1	-0.520	4.26E-35	1.28E-32
TAOK1	17q11.2	-0.520	4.28E-35	1.28E-32
BBX	3q13.12	-0.519	4.94E-35	1.42E-32
INTS2	17q23.2	-0.519	5.98E-35	1.67E-32
ZSCAN29	15q15.3	-0.518	7.38E-35	2.00E-32
PPP1R12A	12q21.2-q21.31	-0.518	8.16E-35	2.18E-32
YLPM1	14q24.3	-0.518	8.69E-35	2.26E-32
DHX33	17p13.2	-0.517	1.03E-34	2.63E-32
PHF3	6q12	-0.516	1.37E-34	3.37E-32
FAM168A	11q13.4	-0.516	1.41E-34	3.41E-32
DPP8	15q22.31	-0.515	1.93E-34	4.56E-32
ADAM10	15q21.3	-0.515	1.98E-34	4.61E-32
BIRC6	2p22.3	-0.515	2.09E-34	4.83E-32
UGGT1	2q14.3	-0.515	2.31E-34	5.26E-32
CBL	11q23.3	-0.515	2.42E-34	5.46E-32
REST	4q12	-0.514	2.96E-34	6.53E-32
TGFBRAP1	2q12.1-q12.2	-0.514	3.22E-34	6.94E-32
N4BP2	4p14	-0.512	5.19E-34	1.09E-31
MDN1	6q15	-0.512	5.28E-34	1.09E-31

Supplementary Table S3 (continued). Top 100 genes with the highest negative expression with *TSPO*.

Correlated gene	Cytoband	Spearman's correlation	p-Value	q-Value
PTPN11	12q24.13	-0.512	6.29E-34	1.29E-31
MGAT5	2q21.2-q21.3	-0.510	1.03E-33	2.07E-31
PDE3A	12p12.2	-0.510	1.04E-33	2.07E-31
TAF1	Xq13.1	-0.510	1.10E-33	2.15E-31
KIRREL1	1q23.1	-0.510	1.17E-33	2.26E-31
CASP8AP2	6q15	-0.510	1.33E-33	2.54E-31
ANKRD40	17q21.33	-0.509	1.81E-33	3.36E-31
KLHL11	17q21.2	-0.508	2.35E-33	4.33E-31
MGA	15q15	-0.508	2.42E-33	4.41E-31
MYO9A	15q23	-0.505	6.12E-33	1.10E-30
SPTBN1	2p16.2	-0.505	7.14E-33	1.27E-30
ZFR	5p13.3	-0.504	8.16E-33	1.41E-30
CKAP5	11p11.2	-0.504	9.53E-33	1.62E-30
REV3L	6q21	-0.503	1.09E-32	1.84E-30
MED13L	12q24.21	-0.503	1.34E-32	2.21E-30
PDPR	16q22.1	-0.502	1.39E-32	2.29E-30
RAB11FIP2	10q26.11	-0.501	1.92E-32	3.11E-30
RASSF8	12p12.1	-0.501	2.66E-32	4.28E-30
UBXN7	3q29	-0.500	2.74E-32	4.36E-30
TBCEL	11q23.3	-0.500	2.77E-32	4.37E-30
PRKDC	8q11.21	-0.500	2.79E-32	4.38E-30
GTF2A1	14q31.1	-0.500	2.87E-32	4.42E-30
MTR	1q43	-0.500	2.87E-32	4.42E-30
ATF2	2q31.1	-0.500	3.10E-32	4.74E-30
TEAD1	11p15.3	-0.500	3.30E-32	4.99E-30
CEP170	1q43	-0.500	3.39E-32	5.07E-30
STK4	20q13.12	-0.499	5.08E-32	7.38E-30
IL6ST	5q11.2	-0.498	5.48E-32	7.85E-30
SEL1L	14q31	-0.498	6.00E-32	8.50E-30
TRIO	5p15.2	-0.498	6.02E-32	8.50E-30
ADNP	20q13.13	-0.496	1.01E-31	1.39E-29
CDC27	17q21.32	-0.496	1.08E-31	1.48E-29
TP53BP1	15q15.3	-0.495	1.69E-31	2.28E-29
SHPRH	6q24.3	-0.494	2.10E-31	2.79E-29
CDK12	17q12	-0.494	2.24E-31	2.96E-29
KDM5A	12p13.33	-0.493	2.59E-31	3.37E-29
ANKRD36BP1	1q24.2	-0.493	2.91E-31	3.76E-29
SLX4IP	20p12.2	-0.492	3.72E-31	4.75E-29
SON	21q22.11	-0.492	3.97E-31	5.04E-29
SMG1	16p12.3	-0.492	4.13E-31	5.21E-29
PCNX1	14q24.2	-0.492	4.37E-31	5.44E-29
PALM2AKAP2	9q31.3	-0.492	4.69E-31	5.81E-29
RANBP2	2q13	-0.491	4.84E-31	5.95E-29
TGOLN2	2p11.2	-0.491	5.45E-31	6.59E-29

Supplementary Table S4. Univariate survival analyses of TSPO expression and clinicopathological features of patients with HNSCC in the TMA patient cohort.

			OS		DSS		DFS	
		TSPO expression	Survival effect HR (95% CI)	p-value	Survival effect HR (95% CI)	p-value	Survival effect HR (95% CI)	p-value
**T**	T1–T2	Low	1	**-**	1	**-**	1	**-**
		High	0.687 (0.476–0.992)	**0.045**	0.469 (0.289–0.760)	**0.002**	0.627 (0.414–0.949)	**0.027**
	T3–T4	Low	1	-	1	-	1	-
		High	0.864 (0.632–1.180)	0.357	0.822 (0.575–1.174)	0.281	1.209 (0.767–1.905)	0.413
**N**	N0	Low	1	-	1	**-**	1	-
		High	0.713 (0.502–1.012)	0.058	0.567 (0.355–0.908)	**0.018**	0.868 (0.553–1.361)	0.537
	N+	Low	1	-	1	**-**	1	-
		High	0.722 (0.526–0.992)	**0.044**	0.658 (0.459–0.941)	**0.022**	0.715 (0.469–1.089)	0.118
**Stage**	0–II	Low	1	-	1	**-**	1	-
		High	0.733 (0.461–1.164)	0.188	0.430 (0.222–0.835)	**0.013**	0.613 (0.365–1.031)	0.065
	III–IV	Low	1	-	1	**-**	1	-
		High	0.764 (0.582–1.003)	0.053	0.722 (0.527–0.988)	**0.042**	0.939 (0.646–1.364)	0.740
**Grade**	G1	Low	1	-	1	-	1	-
		High	0.766 (0.470–1.251)	0.287	0.554 (0.297–1.033)	0.063	0.674 (0.384–1.181)	0.168
	G2	Low	1	-	1	-	1	-
		High	0.629 (0.440–0.899)	**0.011**	0.571 (0.376–0.868)	**0.009**	0.706 (0.451–1.106)	0.128
	G3	Low	1	**-**	1	**-**	1	-
		High	0.772 (0.493–1.209)	0.258	0.667 (0.390–1.139)	0.138	1.029 (0.539–1.965)	0.931

TSPO staining intensity scores of 0–1 were considered low and scores 2–3 as high. Statistical significance was calculated using Cox's proportional hazard model. p-values < 0.05 were considered statistically significant (shown in bold). OS = overall survival, DSS = disease-specific survival, DFS = disease-free survival, HR = hazard ratio, CI = confidence interval.

Supplementary Table S5. Gene overlaps for CP:KEGG, GO:BP, and Hallmark gene sets for genes expressed positively with *TSPO* expression.

Gene symbol	KEGG_**OXIDATIVE_PHOSPHORYLATION** p = 1.22E-17	KEGG_**PARKINSONS_DISEASE** p = 5.07E-16	KEGG_**HUNTINGTONS_DISEASE** p = 8.51E-16	KEGG_**ALZHEIMERS_DISEASE** p = 9.97E-15	KEGG_**RIBOSOME** p = 5.14E-11	KEGG_**CARDIAC_MUSCLE_CONTRACTION** p = 1.04E-9	GOBP_**OXIDATIVE_PHOSPHORYLATION** p = 7.03E-19	GOBP_**CELLULAR_RESPIRATION** p = 2.43E-17	GOBP_**AEROBIC_RESPIRATION** p = 3.84E-17	GOBP_**RESP_ELECTR_TRANSP_CHAIN** p = 1.87E-16	GOBP_**ATP_SYNTH_COUPL_ELECTR_TRANSP** p = 1.04E-15	GOBP_**ORGANONITROGEN_COMP_BIOSYNT_PROC** p = 1.21E-15	GOBP_**ENERGY_DERIVAT_BY_OXIDAT_ORG_COMP** p = 3.11E-15	GOBP_**ELECTRON_TRANSPORT_CHAIN** p = 1.43E-14	GOBP_**GENER_PRECUR_METABOL_AND_ENERGY** p = 7.81E-14	GOBP_**PEPTIDE_BIOSYNTHETIC_PROCESS** p = 9.03E-12	HALLMARK_**OXIDATIVE_PHOSPHORYLATION** p = 2.17E-18	HALLMARK_**ADIPOGENESIS** p = 1.69E-9	HALLMARK_**DNA_REPAIR** p = 1.94E-6
UQCR11	■	■	■	■	■	■	■	■	■	■	■		■	■	■		■	■	
COX4I1	■	■	■	■		■	■	■	■	■	■		■	■	■		■		
COX6A1	■	■	■	■		■	■	■	■	■	■		■	■	■		■	■	
COX7A2	■	■	■	■		■	■	■	■	■	■		■	■	■		■		
COX8A	■	■	■	■		■	■	■	■	■	■		■	■	■		■	■	
UQCRQ	■	■	■	■		■	■	■	■	■	■		■	■	■		■	■	
UQCR10	■	■	■	■		■	■	■	■	■	■		■	■	■		■	■	
NDUFS7	■	■	■	■		■	■	■	■	■	■	■	■	■	■		■		
NDUFA6	■	■	■	■			■	■	■	■	■	■	■	■	■		■		
NDUFA7	■	■	■	■			■	■	■	■	■	■	■	■	■	■	■		
NDUFB7	■	■	■	■			■	■	■	■	■	■	■	■	■		■	■	
ATP5F1D	■	■	■	■			■	■	■			■	■		■		■		
NDUFA11	■						■	■	■			■	■		■				
POLR2F			■														■		■
RPL36					■							■				■			
RPL18A					■							■				■			
RPL34					■							■				■			
RPL37A					■							■				■			
RPS9					■							■				■			
RPS15					■							■				■			
RPS19					■							■				■			
UBA52					■														
ECHS1																	■	■	
TOMM22																	■		

Supplementary Table S5 (continued). Gene overlaps for CP:KEGG, GO:BP, and Hallmark gene sets for genes expressed positively with *TSPO* expression.

Gene symbol	KEGG_OXIDATIVE_PHOSPHORYLATION p = 1.22E-17	KEGG_PARKINSONS_DISEASE p = 5.07E-16	KEGG_HUNTINGTONS_DISEASE p = 8.51E-16	KEGG_ALZHEIMERS_DISEASE p = 9.97E-15	KEGG_RIBOSOME p = 5.14E-11	KEGG_CARDIAC_MUSCLE_CONTRACTION p = 1.04E-9	GOBP_OXIDATIVE_PHOSPHORYLATION p = 7.03E-19	GOBP_CELLULAR_RESPIRATION p = 2.43E-17	GOBP_AEROBIC_RESPIRATION p = 3.84E-17	GOBP_RESP_ELECTR_TRANSP_CHAIN p = 1.87E-16	GOBP_ATP_SYNTH_COUPL_ELECTR_TRANSP p = 1.04E-15	GOBP_ORGANONITROGEN_COMP_BIOSYNT_PROC p = 1.21E-15	GOBP_ENERGY_DERIVAT_BY_OXIDAT_ORG_COMP p = 3.11E-15	GOBP_ELECTRON_TRANSPORT_CHAIN p = 1.43E-14	GOBP_GENER_PRECUR_METABOL_AND_ENERGY p = 7.81E-14	GOBP_PEPTIDE_BIOSYNTHETIC_PROCESS p = 9.03E-12	HALLMARK_OXIDATIVE_PHOSPHORYLATION p = 2.17E-18	HALLMARK_ADIPOGENESIS p = 1.69E-9	HALLMARK_DNA_REPAIR p = 1.94E-6
DDT																		■	
TST																		■	
RBX1																			■
GUK1																			■
NME3																			■
VPS28																			■
EDF1																			■
NDUFA13							■	■	■			■	■		■				
SCO2								■		■			■	■	■				
ATF4												■				■			
COA3												■				■			
UBA52												■				■			
PYM1												■				■			
HSPB1												■				■			
AURKAIP1												■				■			
MRPL41												■				■			
GADD45GIP1												■				■			
MRPS34												■				■			
ATP5MK												■							
NME3												■							
PMM1												■							
GUK1												■							
ILVBL												■							
PGLS															■				

Supplementary Table S6. Gene overlaps for CP:KEGG, GO:BP, and Hallmark gene sets for genes expressed negatively with *TSPO* expression.

Gene symbol	KEGG_**BASAL_TRANSCRIPTION_FACTORS** p = 7.83E-5	KEGG_**UBIQUITIN_MEDIATED_PROTEOLYSIS** p = 3.02E-4	KEGG_**MAPK_SIGNALING_PATHWAY** p = 4.36E-4	KEGG_**ENDOCYTOSIS** p = 9.08E-4	KEGG_**TGF_BETA_SIGNALING_PATHWAY** p = 1.12E-3	GOBP_**PHOSPHORYLATION** p = 6.03E-13	GOBP_**CELL_CYCLE** p = 5.86E-12	GOBP_**CELL_CYCLE_PROCESS** p = 2.44E-11	GOBP_**PROTEIN_CONTAINING_COMPLEX_ORGANIZATION** p = 3.34E-11	GOBP_POSITIVE_**REGULATION_OF_RNA_METABOLIC_PROCESS** p = 4.37E-11	GOBP_REGULATION_**OF_PROTEIN_MODIFICATION_PROCESS** p = 3.72E-10	GOBP_**NUCLEAR_TRANSPORT** p = 3.76E-10	GOBP_**REGULATION_OF_CATALYTIC_ACTIVITY** p = 1.83E-9	GOBP_**CELLULAR_RESPONSE_TO_DNA_DAMAGE_STIMULUS** p = 2.02E-9	GOBP_**POSITIVE_REGULATION_OF_MACROMOLECULE_BIOSYNTHETIC_PROC** p = 2.82E-9	HALLMARK_**MITOTIC_SPINDLE** p = 4.92E-7	HALLMARK_**IL6_JAK_STAT3_SIGNALING** p = 1.16E-3	HALLMARK_**G2M_CHECKPOINT** p = 1.31E-3
TAF1L	■						■	■	■	■					■			
GTF2A1	■																	
TAF1	■					■	■	■	■	■	■			■	■			
SMURF2		■		■	■													
CBL		■		■		■					■		■	■			■	
CDC27		■					■	■								■		■
BIRC6		■				■	■	■					■					
STK4			■			■			■		■	■						
MAP3K2			■			■												
ATF2			■			■	■	■		■	■	■		■	■			
NF1			■			■					■	■	■			■		
TAOK1			■			■	■	■	■		■			■				
ASAP1				■									■					
RAB11FIP2				■									■					
ROCK1					■	■	■	■		■	■		■			■		
ROCK2					■	■	■	■		■	■		■					
SPTBN1						■	■	■	■							■		
TRIO						■										■		
LATS1						■	■	■	■		■		■			■		
CKAP5																■		
IL6ST						■					■						■	
PTPN11						■	■	■	■		■	■		■			■	
ATRX							■	■	■	■				■	■			■
CCNT1						■	■			■	■		■		■			■
CASP8AP2							■						■					■
PRKDC						■	■	■	■	■	■			■	■			
CDK12						■	■			■	■		■		■			
KIRREL1						■			■		■							
PIK3C2A						■			■									
TTBK2						■			■									
ADAM10						■			■									
ADNP						■					■		■					
RANBP2						■						■	■					

Supplementary Table S6 (continued). Gene overlaps for CP:KEGG, GO:BP, and Hallmark gene sets for genes expressed negatively with *TSPO* expression.

Gene symbol	KEGG_BASAL_TRANSCRIPTION_FACTORS p = 7.83E-5	KEGG_UBIQUITIN_MEDIATED_PROTEOLYSIS p = 3.02E-4	KEGG_MAPK_SIGNALING_PATHWAY p = 4.36E-4	KEGG_ENDOCYTOSIS p = 9.08E-4	KEGG_TGF_BETA_SIGNALING_PATHWAY p = 1.12E-3	GOBP_PHOSPHORYLATION p = 6.03E-13	GOBP_CELL_CYCLE p = 5.86E-12	GOBP_CELL_CYCLE_PROCESS p = 2.44E-11	GOBP_PROTEIN_CONTAINING_COMPLEX_ORGANIZATION p = 3.34E-11	GOBP_POSITIVE_REGULATION_OF_RNA_METABOLIC_PROCESS p = 4.37E-11	GOBP_REGULATION_OF_PROTEIN_MODIFICATION_PROCESS p = 3.72E-10	GOBP_NUCLEAR_TRANSPORT p = 3.76E-10	GOBP_REGULATION_OF_CATALYTIC_ACTIVITY p = 1.83E-9	GOBP_CELLULAR_RESPONSE_TO_DNA_DAMAGE_STIMULUS p = 2.02E-9	GOBP_POSITIVE_REGULATION_OF_MACROMOLECULE_BIOSYNTHETIC_PROC p = 2.82E-9	HALLMARK_MITOTIC_SPINDLE p = 4.92E-7	HALLMARK_IL6_JAK_STAT3_SIGNALING p = 1.16E-3	HALLMARK_G2M_CHECKPOINT p = 1.31E-3
SMG1						■						■		■				
MED1							■	■	■	■		■			■			
TP53BP1							■	■	■	■				■	■			
CKAP5							■	■	■									
PPP1R12A							■	■		■	■	■	■		■			
ERCC4							■	■					■	■				
PDE3A							■	■					■					
SON							■	■										
RIF1							■				■			■				
DHX33									■	■					■			
GTF2A1									■	■					■			
TEAD1									■	■					■			
MDN1									■			■						
SHPRH									■					■				
TGFBRAP1									■									
TBCEL									■									
TRIM44										■	■				■			
REST										■			■		■			
TCF12										■					■			
ASXL2										■					■			
MED13										■					■			
ZNF281										■					■			
KDM5A										■					■			
MGA										■					■			
MGAT5											■		■					
RGPD4												■						
RGPD3												■						
LIMS1													■					
MYO9A													■					
PDPR													■					
ERCC6L2														■				
FAM168A														■				
REV3L														■				

Supplementary Table S7. Abbreviations for cancer types listed in Figure 4D.

**Abbreviation**	**Cancer**
ACC	Adrenocortical carcinoma
BLCA	Bladder urothelial carcinoma
BRCA	Breast invasive carcinoma
CESC	Cervical and endocervical cancers
CHOL	Cholangiocarcinoma
COAD	Colon adenocarcinoma
ESCA	Esophageal carcinoma
KICH	Kidney chromophobe
KIRC	Kidney renal clear cell carcinoma
KIRP	Kidney renal papillary cell carcinoma
LIHC	Liver hepatocellular carcinoma
LUAD	Lung adenocarcinoma
LUSC	Lung squamous cell carcinoma
MESO	Mesothelioma
OV	Ovarian serous cystadenocarcinoma
PAAD	Pancreatic adenocarcinoma
PCPG	Pheochromocytoma and paraganglioma
PRAD	Prostate adenocarcinoma
READ	Rectum adenocarcinoma
SARC	Sarcoma
SKCM	Skin cutaneous melanoma
STAD	Stomach adenocarcinoma
THCA	Thyroid carcinoma
UCEC	Uterine corpus endometrial carcinoma
UCS	Uterine carcinosarcoma
UVM	Uveal melanoma

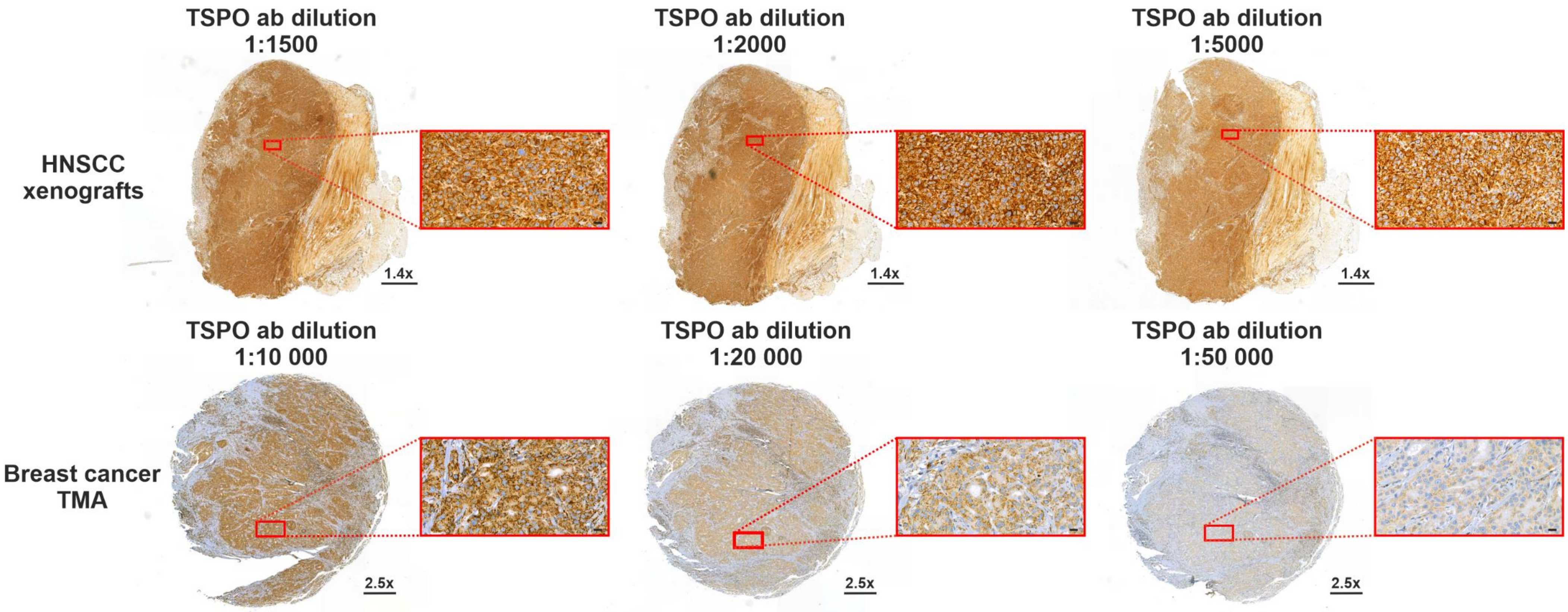

Supplementary Fig. S1 TSPO IHC optimization. TSPO IHC staining with different antibody (monoclonal rabbit TSPO antibody, Abcam ab109497) dilutions were performed to find the optimal antibody dilution for TMA IHC stainings. Stainings were done with HNSCC xenograft tumors and breast cancer TMAs. 1:50 000 dilution was found to be the most optimal for stainings. Scale bars for xenograft tumors: 1000 µm (whole section) and 20 µm (inset). Scale bars for breast cancer TMA sections: 500 µm (whole section) and 20 µm (inset).

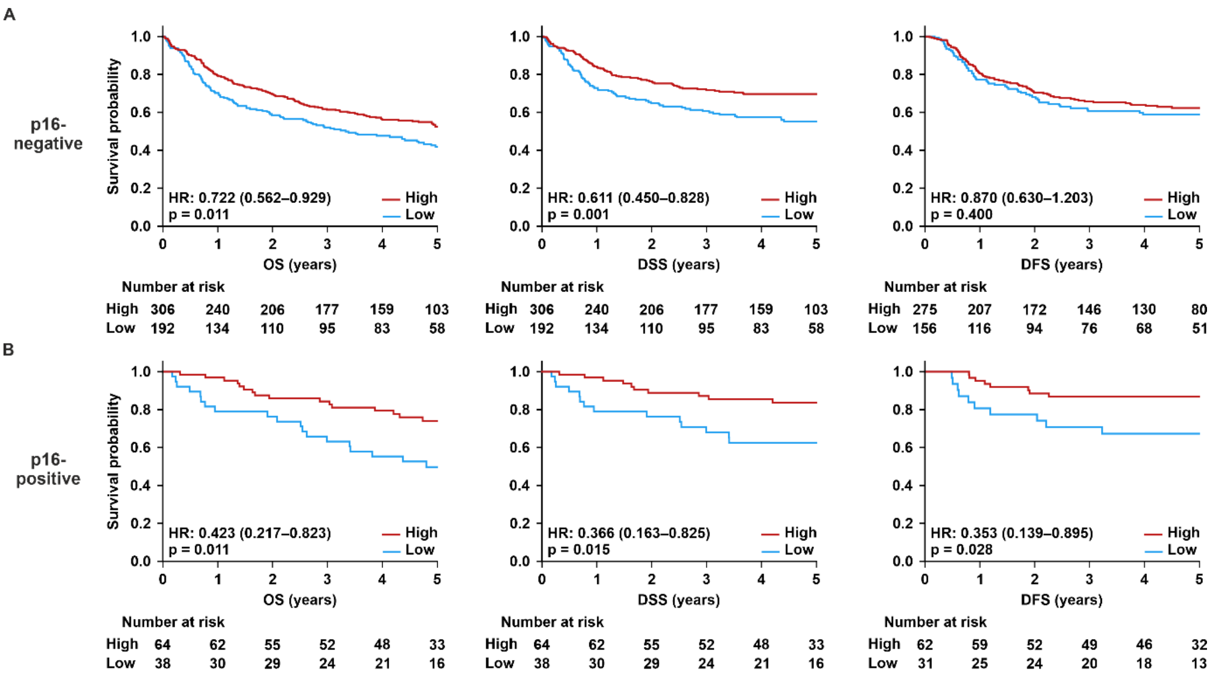

Supplementary Fig. S2 Effect of p16 and TSPO expression on survival in the TMA cohort. Prognostic trends with HR (95% CI) for 5-year overall survival (OS), disease-specific survival (DSS), and disease-free survival (DFS) divided into low and high (staining intensity scores of 0–1 and 2–3, respectively) TSPO tumor expression in patients with (**A**) p16-negative or (**B**) p16-positive tumors. Statistical significance was calculated using Cox's proportional hazard model. p-values < 0.05 were considered statistically significant.

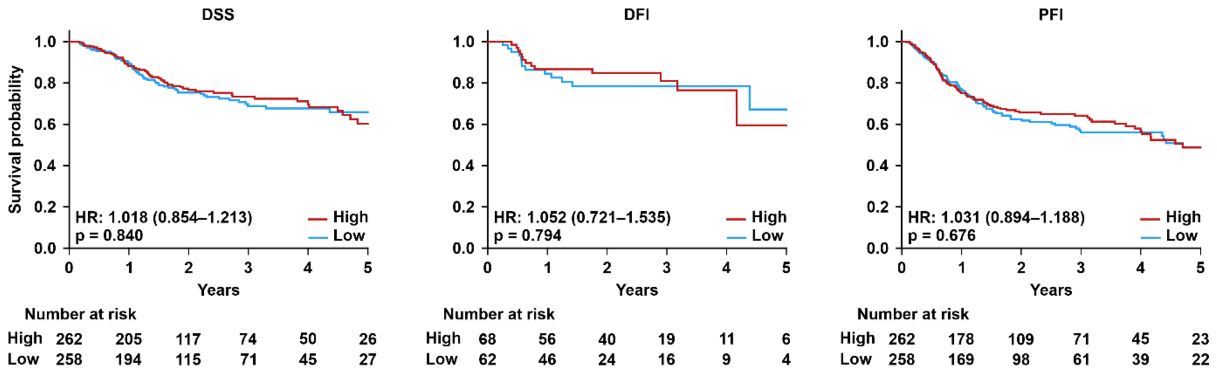

Supplementary Fig. S3 Effect of *TSPO* expression on survival in the TCGA HNSCC cohort. Prognostic trends with HR (95% CI) for 5-year disease-specific survival (DSS), disease-free interval (DFI), and progression-free interval (PFI) in low (below median) and high (equal to or above median) *TSPO* expression in all patients. Statistical significance was calculated using Cox's proportional hazard model. p-values < 0.05 were considered statistically significant.

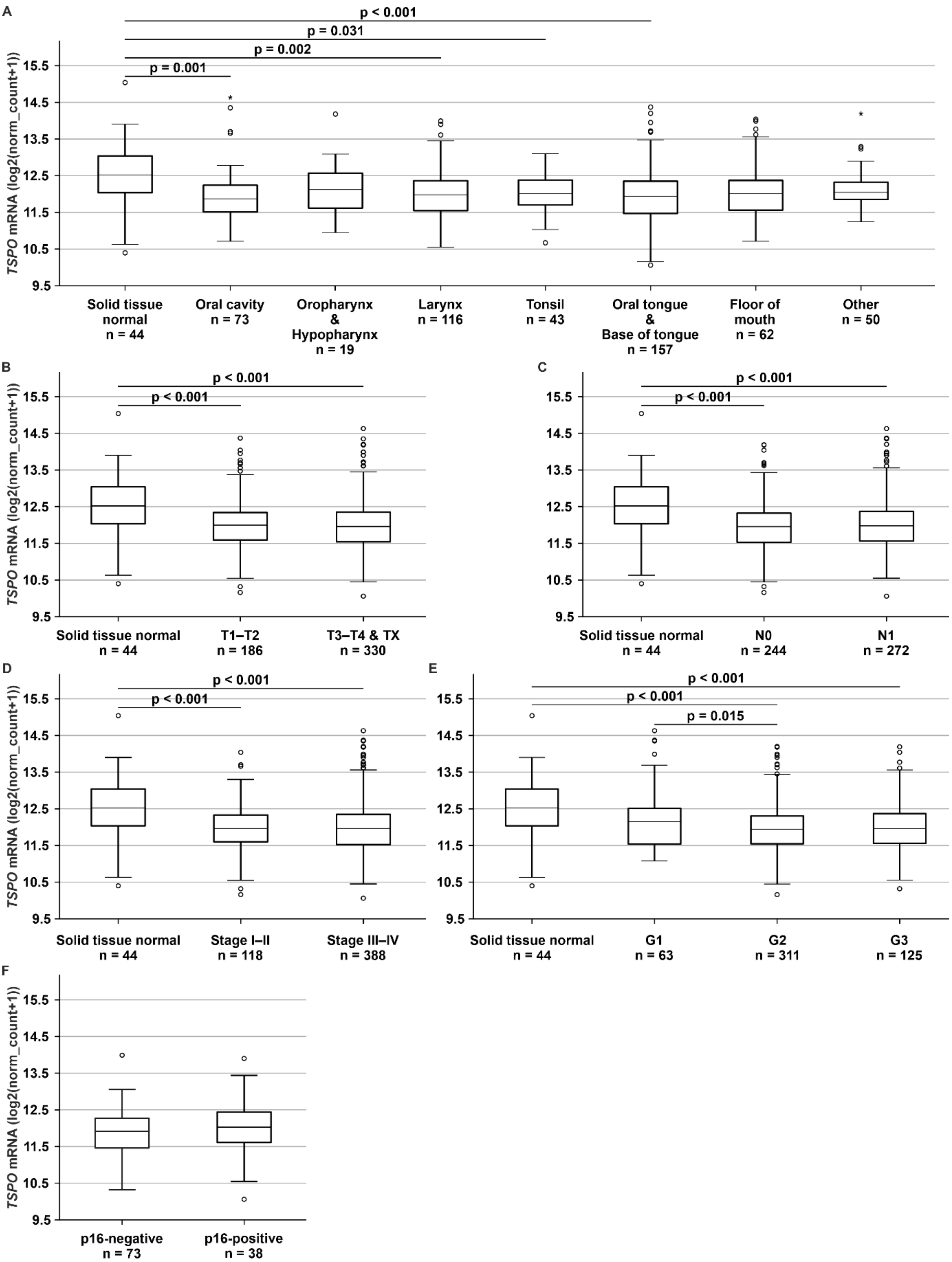

Supplementary Fig. S4 *TSPO* expression in clinicopathological subgroups from the TCGA HNSCC cohort. Boxplots visualizing *TSPO* expression according to (**A**) primary tumor site, (**B**) T classification, (**C**) N classification, (**D**) overall stage, (**E**) grade, and **(F**) p16 status. The box ranges from Q1 (first quartile) to Q3 (third quartile) of the median distribution, and the whiskers represent the range between minimum and maximum. Dots represent outlier values. Independent samples t-test (for comparing two groups) or one-way ANOVA with Bonferroni post hoc tests (for comparing more than two groups) was used to analyze differences in *TSPO* expression. p-values < 0.05 were considered statistically significant.

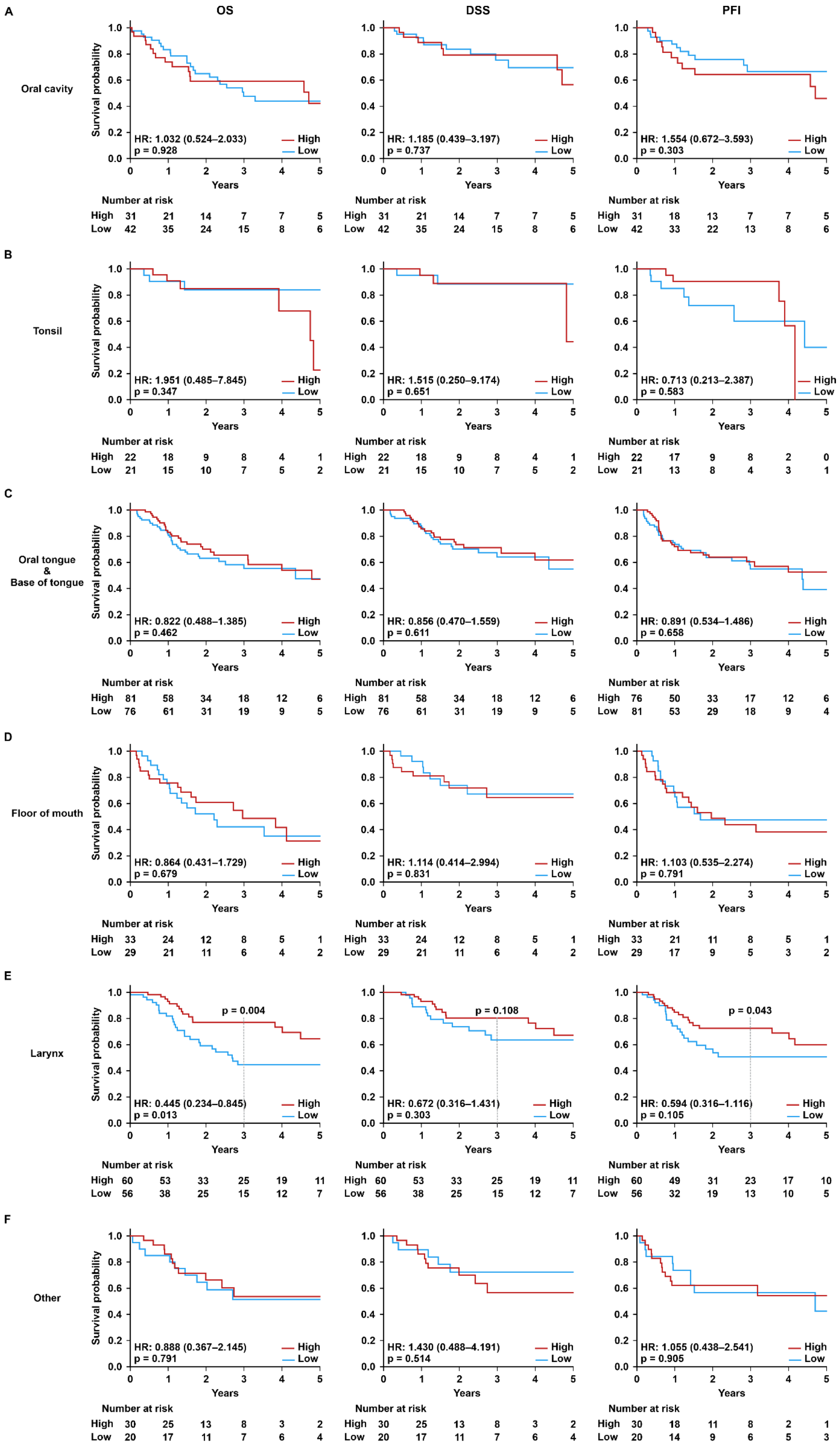
A
OS
DSS
PFI
Oral cavity
Survival probability
Years
HR: 1.032 (0.524–2.033)
p = 0.928
HR: 1.185 (0.439–3.197)
p = 0.737
HR: 1.554 (0.672–3.593)
p = 0.303
High
Low
Number at risk
High 31 21 14 7 7 5
Low 42 35 24 15 8 6
High 31 21 14 7 7 5
Low 42 35 24 15 8 6
High 31 18 13 7 7 5
Low 42 33 22 13 8 6
B
Tonsil
HR: 1.951 (0.485–7.845)
p = 0.347
HR: 1.515 (0.250–9.174)
p = 0.651
HR: 0.713 (0.213–2.387)
p = 0.583
High 22 18 9 8 4 1
Low 21 15 10 7 5 2
High 22 18 9 8 4 1
Low 21 15 10 7 5 2
High 22 17 9 8 2 0
Low 21 13 8 4 3 1
C
Oral tongue
&
Base of tongue
HR: 0.822 (0.488–1.385)
p = 0.462
HR: 0.856 (0.470–1.559)
p = 0.611
HR: 0.891 (0.534–1.486)
p = 0.658
High 81 58 34 18 12 6
Low 76 61 31 19 9 5
High 81 58 34 18 12 6
Low 76 61 31 19 9 5
High 76 50 33 17 12 6
Low 81 53 29 18 9 4
D
Floor of mouth
HR: 0.864 (0.431–1.729)
p = 0.679
HR: 1.114 (0.414–2.994)
p = 0.831
HR: 1.103 (0.535–2.274)
p = 0.791
High 33 24 12 8 5 1
Low 29 21 11 6 4 2
High 33 24 12 8 5 1
Low 29 21 11 6 4 2
High 33 21 11 8 5 1
Low 29 17 9 5 3 2
E
Larynx
p = 0.004
p = 0.108
p = 0.043
HR: 0.445 (0.234–0.845)
p = 0.013
HR: 0.672 (0.316–1.431)
p = 0.303
HR: 0.594 (0.316–1.116)
p = 0.105
High 60 53 33 25 19 11
Low 56 38 25 15 12 7
High 60 53 33 25 19 11
Low 56 38 25 15 12 7
High 60 49 31 23 17 10
Low 56 32 19 13 10 5
F
Other
HR: 0.888 (0.367–2.145)
p = 0.791
HR: 1.430 (0.488–4.191)
p = 0.514
HR: 1.055 (0.438–2.541)
p = 0.905
High 30 25 13 8 3 2
Low 20 17 11 7 6 4
High 30 25 13 8 3 2
Low 20 17 11 7 6 4
High 30 18 11 8 2 1
Low 20 14 9 6 5 3

Supplementary Fig. S5 *TSPO* expression and site-specific survival analyses from the HNSCC TCGA cohort. Prognostic trends with HR (95% CI) for 5-year overall survival (OS), disease-specific survival (DSS), and progression-free interval (PFI) for *TSPO* expression in (**A**) oral cavity, (**B**) tonsil, (**C**) oral tongue and base of tongue, (**D**) floor of mouth, (**E**) larynx, and (**F**) other tumor sites. *TSPO* expression was divided into low (expression below median) and high (expression equal to or above median). Statistical significance was calculated using Cox's proportional hazard model. p-values < 0.05 were considered statistically significant.

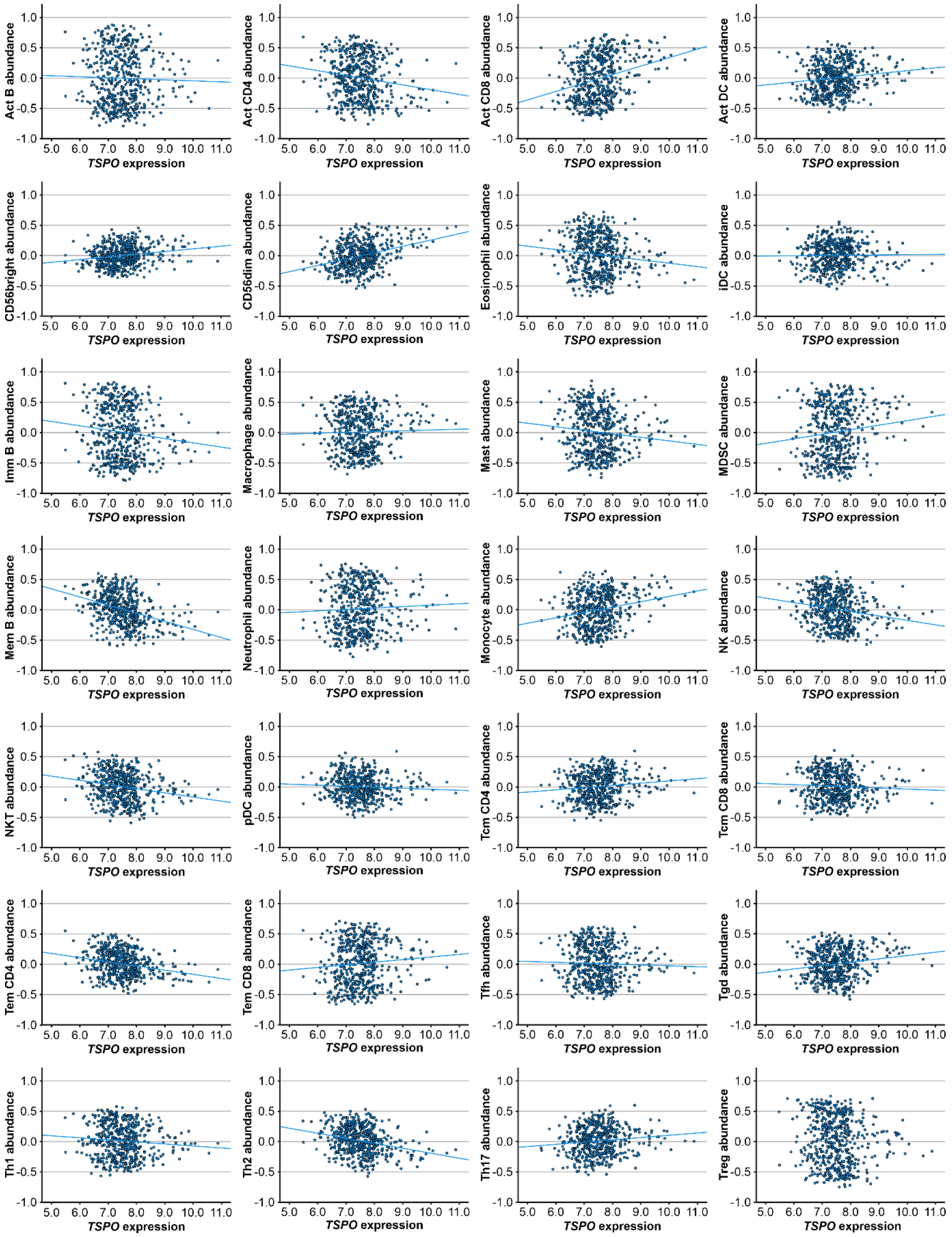

Supplementary Fig. S6 Scatter plots of immune cell abundance in relation to *TSPO* expression in HNSCC. The heatmap is shown in Figure 4D. Act = activated, IDC = interstitial dendritic cell, Imm = immature, MDSC = myeloid-derived suppressor cell, Mem = memory, NK = natural killer cell, NKT = natural killer T cell, pDC = plasmacytoid dendritic cell, Tcm = central memory cell, Tem = effector memory cell, Tfh = T follicular helper cell, Tgd = gamma delta T cell, Th1 = type 1 T helper cell, Th2 = type 2 T helper cell, Th17 = type 17 T helper cell, Treg = regulatory T cell.

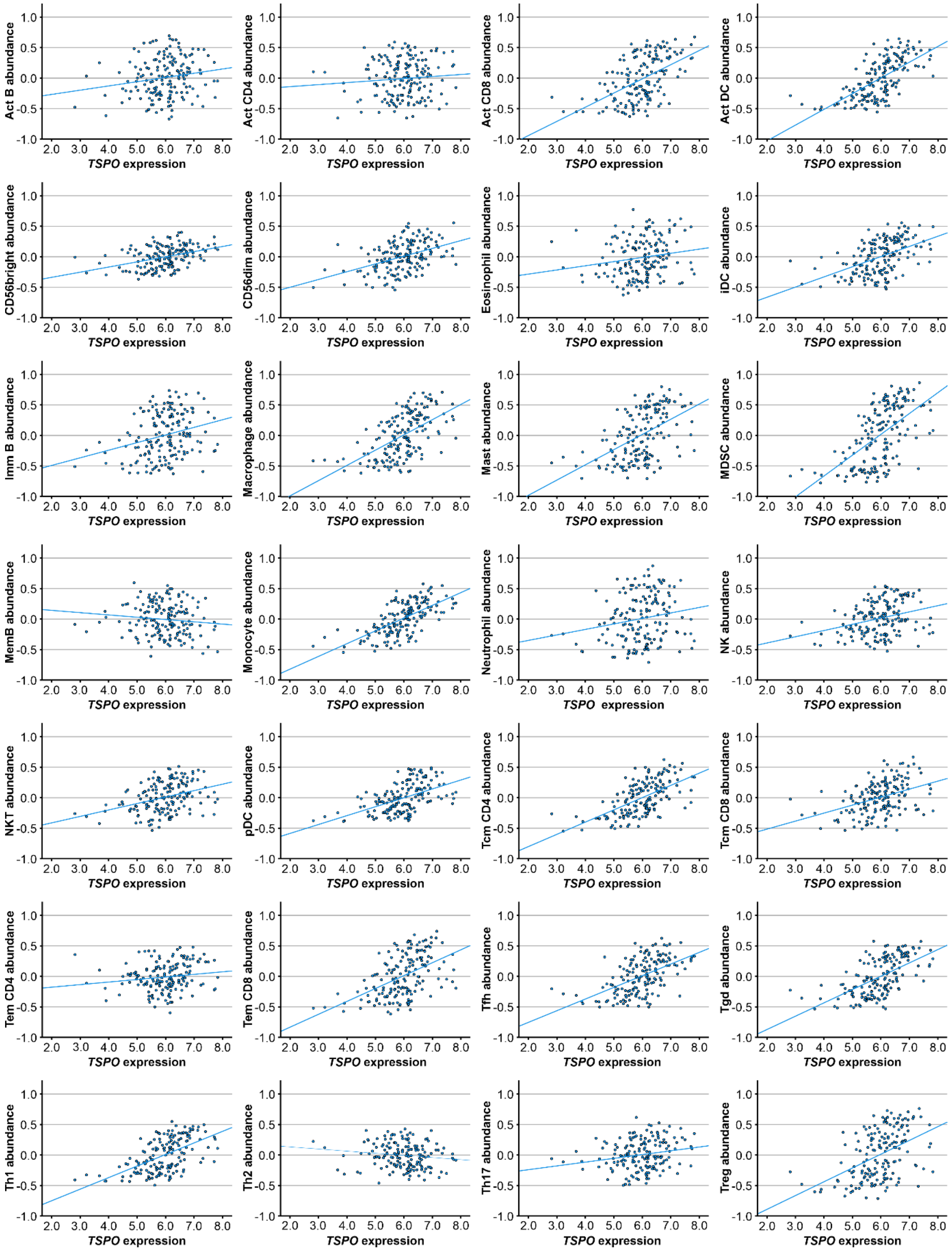

Supplementary Fig. S7 Scatter plots of immune cell abundance in relation to *TSPO* expression in glioblastoma (GBM). The heatmap is shown in Figure 4D. Act = activated, IDC = interstitial dendritic cell, Imm = immature, MDSC = myeloid-derived suppressor cell, Mem = memory, NK = natural killer cell, NKT = natural killer T cell, pDC = plasmacytoid dendritic cell, Tcm = central memory cell, Tem = effector memory cell, Tfh = T follicular helper cell, Tgd = gamma delta T cell, Th1 = type 1 T helper cell, Th2 = type 2 T helper cell, Th17 = type 17 T helper cell, Treg = regulatory T cell.

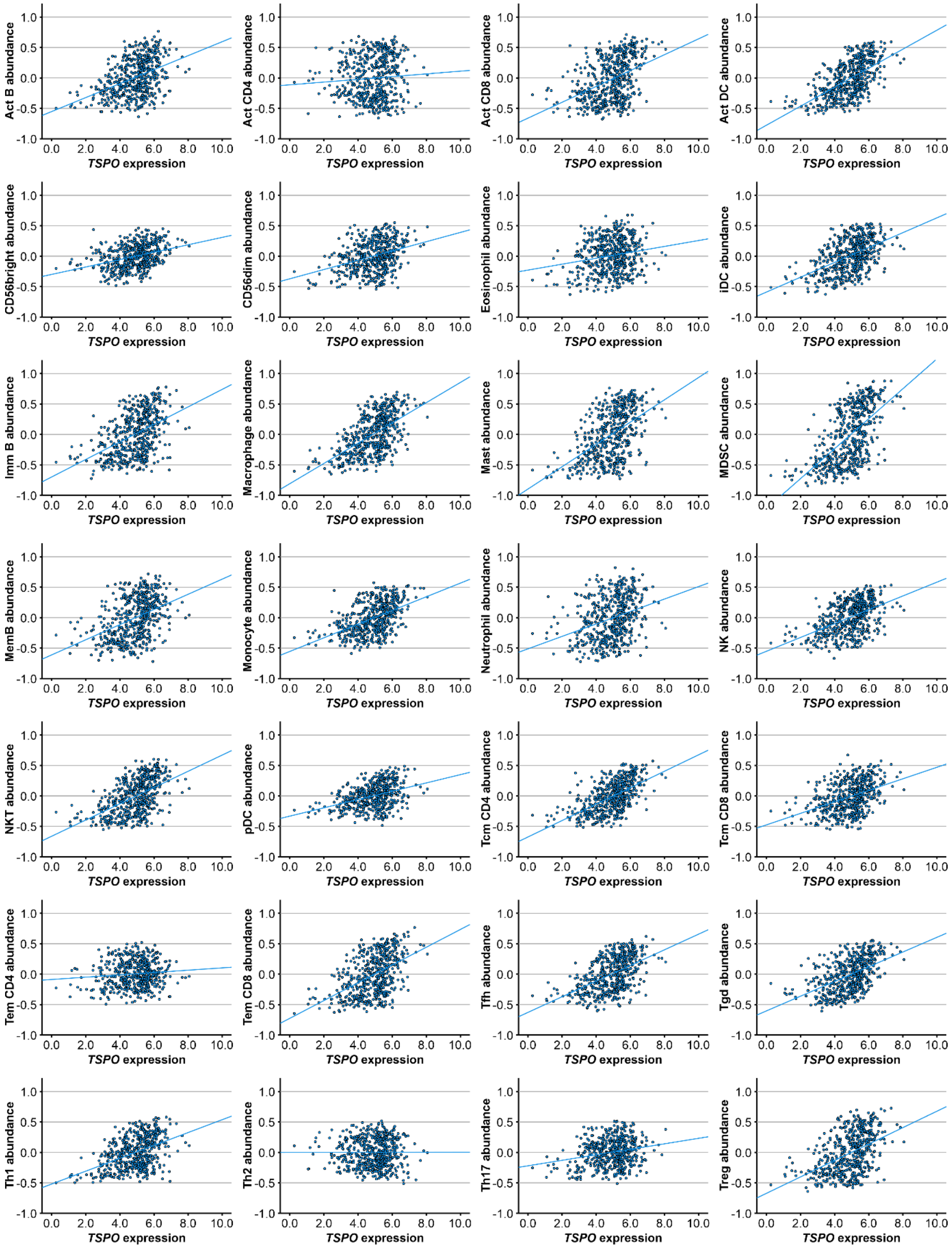

Supplementary Fig. S8 Scatter plots of immune cell abundance in relation to *TSPO* expression in lower grade glioma (LGG). The heatmap is shown in Figure 4D. Act = activated, IDC = interstitial dendritic cell, Imm = immature, MDSC = myeloid-derived suppressor cell, Mem = memory, NK = natural killer cell, NKT = natural killer T cell, pDC = plasmacytoid dendritic cell, Tcm = central memory cell, Tem = effector memory cell, Tfh = T follicular helper cell, Tgd = gamma delta T cell, Th1 = type 1 T helper cell, Th2 = type 2 T helper cell, Th17 = type 17 T helper cell, Treg = regulatory T cell.

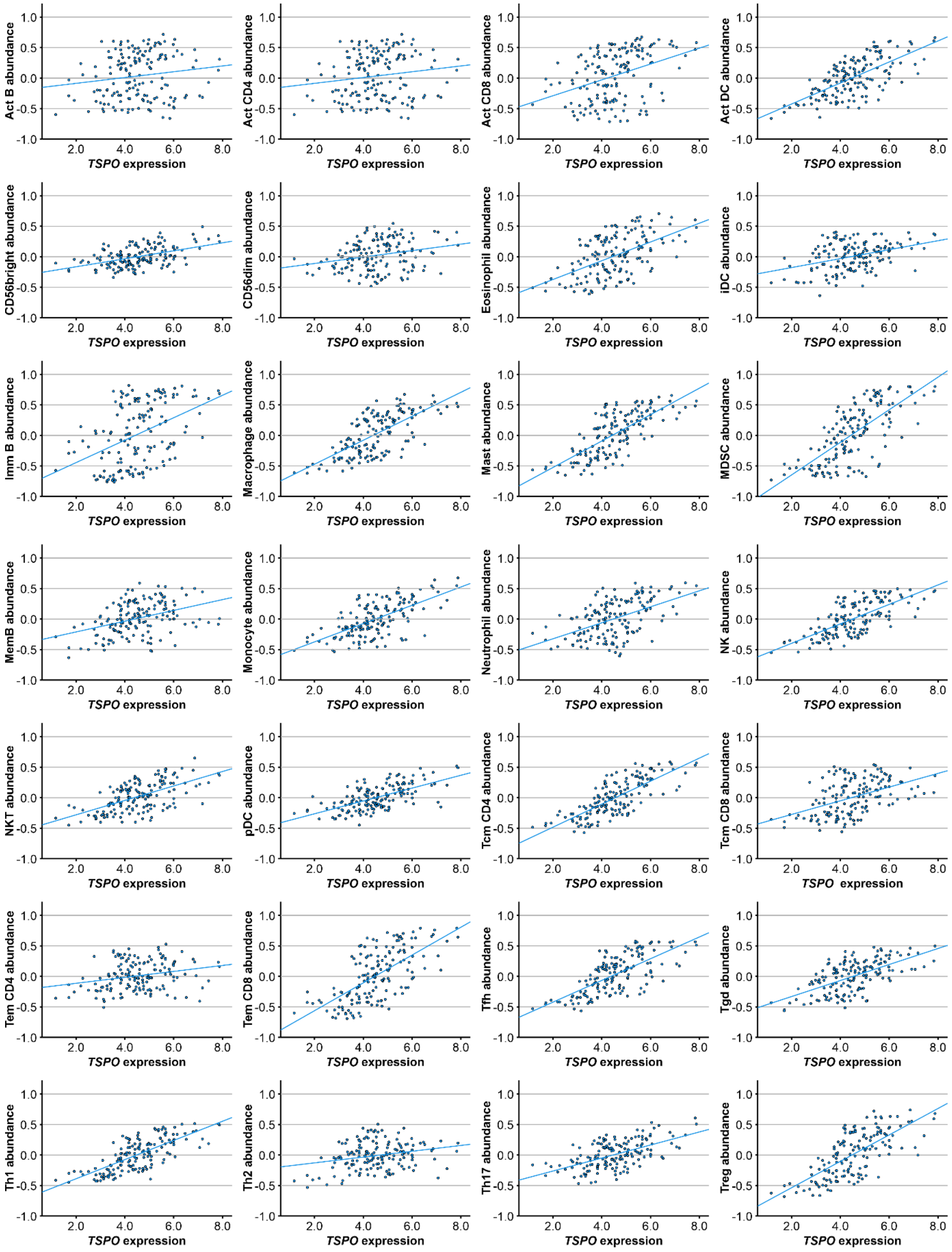

Supplementary Fig. S9 Scatter plots of immune cell abundance in relation to *TSPO* expression in testicular germ cell tumors (TGCT). The heatmap is shown in Figure 4D. Act = activated, IDC = interstitial dendritic cell, Imm = immature, MDSC = myeloid-derived suppressor cell, Mem = memory, NK = natural killer cell, NKT = natural killer T cell, pDC = plasmacytoid dendritic cell, Tcm = central memory cell, Tem = effector memory cell, Tfh = T follicular helper cell, Tgd = gamma delta T cell, Th1 = type 1 T helper cell, Th2 = type 2 T helper cell, Th17 = type 17 T helper cell, Treg = regulatory T cell.
